# Association between dairy intake and multiple health outcomes: a scoping review of systematic reviews and meta-analyses

**DOI:** 10.1038/s41430-025-01639-5

**Published:** 2025-07-26

**Authors:** Saskia Akyil, Stefanie Winkler, Dorothy Meyer, Eva Kiesswetter, Martin Kussmann, Lukas Schwingshackl, Hans Hauner

**Affiliations:** 1 Institute of Nutritional Medicine, Else Kröner-Fresenius-Center for Nutritional Medicine, TUM School of Medicine and Health, Technical University of Munich, Munich, Germany; 2 Institute for Evidence in Medicine, Medical Center - University of Freiburg, Faculty of Medicine, University of Freiburg, Freiburg, Germany; 3 Competence Center of Nutrition (KErn) at the Bavarian State Research Center for Agriculture, Freising, Germany; 4 Kussmann Biotech GmbH, Nordkirchen, Germany

**Keywords:** Risk factors, Scientific community

## Abstract

Food-based dietary guidelines acknowledge non-fortified dairy foods as a source of multiple essential vitamins and minerals as well as high-quality protein. Considering the cultural significance of dairy foods in our diet and the increasing prevalence of non-communicable diseases, it is essential to continuously evaluate the entirety of data regarding the impact of dairy consumption on various health-related outcomes. A systematic literature search was performed in three databases: Medline, Embase, and Web of Science. Systematic reviews published between January 2014 and February 2024 based on randomized controlled trials (RCTs), prospective cohort studies, case-control studies, and/or cross-sectional studies in adults, focusing on the consumption of bovine dairy products were evaluated for inclusion. Reports from the World Cancer Research Fund on selected cancer outcomes were also included in this review. We identified 95 reports encompassing five dairy exposure categories on 29 different health outcomes. Out of 281 associations identified, 37.7% linked dairy consumption to a reduced risk, while 48.0% showed no association with disease risk. Inconclusive results were found in 10.0% of the associations, and 4.3% indicated an increased risk of adverse health outcomes. Overall, the evidence suggests that consuming dairy is not associated with an increased risk of non-communicable diseases or mortality. In fact, it may moderately reduce the risk of several health outcomes, including adverse cardiovascular outcomes and certain cancers such as bladder, breast, colorectal, liver, oral, and ovarian. Some studies have also linked dairy consumption to improved body composition, lower rates of type 2 diabetes, and better bone health.

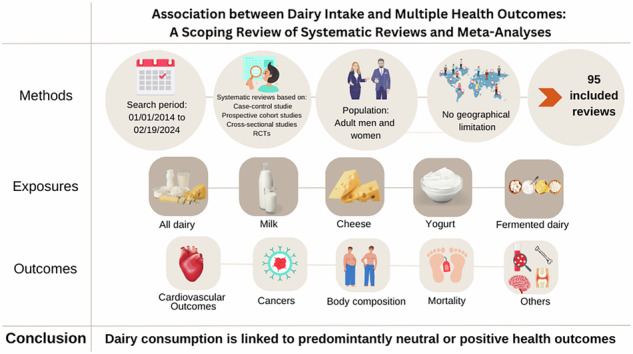

## Introduction

Dairy products have constituted a significant component of the human diet for ~8000 years and are characterized as a distinct food group in many food-based dietary guidelines worldwide [1, 2]. These guidelines acknowledge non-fortified dairy foods as a source of high-quality protein, minerals including calcium, phosphorus, magnesium, selenium, zinc, iodine, and vitamins A, B1, B2, and B12 [2, 3]. Most dietary guidelines recommend a daily intake of 2–3 dairy servings, including low-fat milk, yogurt, and cheese, to fulfill nutrient requirements and enhance dietary quality [4, 5]. Despite these recommendations, research consistently indicates that most individuals fall short of the suggested daily intake [6, 7].

In recent years, there has been a growing interest in exploring the associations between dairy consumption and health outcomes. Systematic reviews indicate that dairy is associated with a reduced risk of various noncommunicable diseases [8]. Despite evidence that dairy intake offers several health benefits, some dairy products are high in saturated fatty acids which have been linked to adverse health effects and are therefore limited in many dietary guidelines [9].

Although less frequently highlighted in food-based dietary guideline messaging, it has been suggested that fermented dairy products such as yogurt and kefir provide health benefits that extend beyond their nutritive components. For instance, these products contain probiotics which may positively influence oral health, gut health, and overall immune function [10–13]. Considering the cultural significance of dairy in our diet and the increasing prevalence of noncommunicable diseases, it is essential to continuously evaluate the entirety of data regarding the impact of dairy consumption on various health-related outcomes.

Scoping reviews of systematic reviews map the existing evidence and identify research gaps on a broad topic. While they appear similar, umbrella reviews and scoping reviews of systematic reviews are different in scope and quality appraisal. Scoping reviews are broader and more exploratory than umbrella reviews and are intended to provide a descriptive overview rather than a synthesis of results. The objective of this scoping review was to summarize the research landscape on associations between dairy consumption and noncommunicable disease outcomes in adults. Specifically, we investigated cardiovascular health outcomes, various cancer types, body composition, mortality, and other health outcomes including type 2 diabetes mellitus (T2DM), bone and joint health, and cognitive outcomes. Although there are numerous systematic reviews (SR) on the topic, we are unaware of any scoping review that investigates all types of dairy and its effects on a range of health outcomes.

## Methods

### Protocol and registration

The protocol for this scoping review was established before the beginning of the study and was registered on February 19, 2024, on Open Science Framework (Available at: 10.17605/OSF.IO/YQWVK) [14]. We have followed the PRISMA extension for scoping reviews in composing this review [15].

### Search strategy

A systematic literature search was performed on February 19, 2024, in three databases: Ovid Medline, Ovid Embase, and Web of Science. The search strategy was developed for Medline by an experienced information specialist and adapted for Embase and Web of Science (SA). It comprised two main themes: dairy intake in the adult population and health outcomes, and was restricted to systematic reviews in English, German, French, and Spanish. The full details of the search strategy are available in the supplemental file.

In addition to the articles identified through the database searches, all available reports from the World Cancer Research Fund (WCRF) [16] that addressed dairy consumption were included in the scoping review.

### Selection of studies

Inclusion and exclusion criteria were created following the Population, Concept, Context framework proposed by the Joanna Briggs Institute (JBI) [17].

#### Inclusion criteria

SR or meta-analyses (MA) fulfilling the following criteria were included in the scoping review: i) publication date between January 1st, 2014 and February 19, 2024, ii) studies based on randomized controlled trials (RCTs), prospective cohort studies, case-control studies, and cross-sectional studies iii) studies involving primarily adults as study population, iv) studies focusing on exposure to cow’s milk and/or other dairy product consumption in any amount, v) publications reporting any of the following outcomes were included: cardiovascular outcomes (cardiovascular disease (CVD), coronary heart disease (CHD), stroke, and hypertension), cancer types (bladder, breast, colorectal, corpus uteri, esophagus, leukemia, liver, lung, non-Hodgkin lymphoma, oral, ovarian, pancreatic, prostate, stomach/gastric, “combined cancer” as defined by individual reports), body composition (weight gain, overweight, and obesity), all-cause mortality and cardiovascular mortality, and other outcomes (T2DM, bone and joint health, cognitive outcomes). Outcomes were chosen on the basis of three factors: I) establishment as a diet-influenced non-communicable disease or mortality; II) for cancer outcomes, selection of the 20 cancer types with the highest global incidence based on the WCRF [16]; and III) existence of at least one systematic review and/or meta-analysis on the outcome that also meets all other inclusion criteria. All papers—systematic reviews, meta-analyses, and WCRF reviews will henceforth be referred to as “reports”.

#### Exclusion criteria

Reports were excluded if they met one or more of the following criteria: I) umbrella review, scoping review, and/or systematic reviews or meta-analyses based on in-vitro/animal experiments, II) reports involving infants, children, and adolescents as participants (exception: exposure started in childhood but outcome was assessed in adulthood), III) reports including non-bovine dairy products (sheep, goats, buffaloes, camels, humans, plant-based, formula products), nutritional supplements and dairy products that are explicitly fortified with calcium and/or other nutrients. IV) reports that looked exclusively at risk factors for the disease outcomes of interest.

### Selection process of sources of evidence

All identified reports were uploaded to Zotero 6.0.36 [18] in separate libraries and then imported into the basic version of Rayyan systematic review software (https://www.rayyan.ai/) for the deletion of duplicates, title, and abstract screening [19]. Rayyan’s duplicate tool identified suspected duplicates which were then manually resolved. Title and abstract screening were independently conducted by two reviewers (SA, SW) to exclude references that did not meet the inclusion criteria. Full-text publications of all potentially relevant records were obtained and uploaded to Zotero. At the full text level, reasons for exclusion were recorded. Two reviewers independently evaluated half of the full-text articles. A minimum of 10% of the articles were cross-checked. Discrepancies between reviewers were addressed through discussion; a third reviewer (DM) was consulted to resolve remaining questions.

### Data extraction

An extraction template was pretested on a subset of five reports. Elicit software was utilized to systematically extract relevant data points from the study texts [20]. Extracted data was subsequently examined in detail by two reviewers, who enhanced and corrected the information. The following data were extracted: author, publication year, design and number of included studies, participant counts, study regions, intervention/exposure, comparison parameter, included health outcomes, direction of effect or association, databases searched, search date range, inclusion of sex stratification. The full details of the extraction tables are available in Supplemental Table A. WCRF Continuous Update Project reports present findings in a standardized way. Their criteria for grading evidence for cancer prevention include convincing (strong evidence), probable (strong evidence), limited—suggestive, limited—no conclusion, and substantial effect on risk unlikely (strong evidence). Further information about WCRF grading evidence criteria can be found in the appendices of WCRF reports [21]. For this study, we charted WCRF reports as follows: convincing and strong evidence were charted as stated (decreases risk/increases risk), and limited evidence was charted as neutral. The WCRF extraction table can be found in Supplemental Table B.

### Data categorization and presentation

Table 1 was constructed to present associations between dairy products and all included health outcomes. The numbers in each cell represent the count of SR and/or MA that reported either evidence of a decreased risk, no association, increased risk, or inconclusive evidence (different types of analyses within the report yielded different findings) of the respective dairy product on each health outcome. Several included reports examined multiple health outcomes and/or exposures, resulting in some reports being referenced in multiple cells. Therefore, the total number of “reduced risk,” “no association,” “increased risk,” and “inconclusive” associations may be higher than the number of included reports. Table 2 was created to present subgroup results on the associations of full-fat versus reduced-fat dairy with health outcomes included in some reports.

**Table 1:**
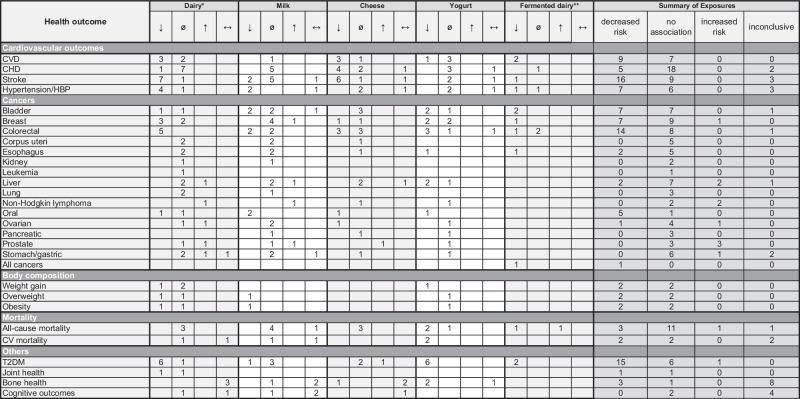
Number of references found for each of the possible associations between dairy products and health outcomes.

”↓“= evidence of decreased risk, “ø”= evidence of no association, “↑“= evidence of increased risk, “↔“= inconclusive evidence; *Studies that looked at overall dairy consumption **Studies that looked at overall fermented dairy consumption; WCRF Continuous Update Reports are reported as follows: “limited-suggestive evidence”: “ø”= evidence of a neutral effect due to being too limited to permit convincing judgment. “Probable decreased risk”: “↓“= evidence of decreased risk.

*CVD* cardiovascular disease, *CHD* coronary heart disease, *HBP* high blood pressure, *CV* cardiovascular, *T2DM* type 2 diabetes mellitus.

**Table 2:** High-fat vs. low-fat milk and dairy subgroup analyses in included reports.

Health Outcome	Author, year	Whole milk	Reduced-fat milk	Full-fat dairy	Reduced-fat dairy
**Cardiovascular outcomes**					
**CVD**	Guo et al. [32]	-	-	no association	no association
**CHD**	Alexander et al. [29]	-	-	no association	inverse association
	Bechthold et al. [37]	-	-	no association	no association
	Gholami et al. [33]	-	-	no association	no association
	Guo et al. [32]	-	-	no association	no association
	Jakobsen et al. [38]	increased association	no association	-	-
	Qin et al. [31]	-	-	inverse association	no association
**Stroke**	Alexander et al. [29]	-	-	inverse association	inverse association
	Bechthold et al. [37]	-	-	no association	no association
	Chen et al. [9]	-	-	inverse association	inverse association
	De Goede et al. [36]	increased association	no association	-	inverse association
	Gholami et al. [33]	-	-	no association	inverse association
	Hu et al. [40]	-	-	no association	inverse association
	Qin et al. [31]	-	-	-	inverse association
**Hypertension/HBP**	Chen et al. [9]	-	-	-	inverse association
	Feng et al. [41]	-	-	no association	inverse association
	Heidari et al. [42]	-	-	-	inverse association
	Kiesswetter et al. [17]^a^	-	-	protective effect^e^	protective effect^e^
	Schwingshackl et al. [43]	-	-	no association	no association
**Cancers**					
**Breast**	Chen et al. [51]	no association	no association	-	-
	Gil et al. [52]	no association	no association	-	-
	He et al. [55]	-	-	no association	inverse association^b^
	Zang et al. [54]	-	-	no association	inverse association
**Colorectal**	Alegria-Lertxundi et al. [63]	no association	no association	no association	no association
**Colorectal**	Barrubes et al. [57]	no association	inverse association	-	no association
	Schwingshackl et al. [61]	-	-	inverse association	inverse association
**Ovarian**	Liao et al. [81]	increased association	inverse association^c^	-	-
	Liu et al. [82]	no association	no association	-	-
**Prostate**	Zhao et al. [85]	inverse association	no association	-	-
**Body composition**					
**Overweight**	Feng et al. [41]	-	-	inverse association	-
**Obesity**	Feng et al. [41]	-	-	inverse association	-
**Mortality**					
**All-cause mortality**	Guo et al. [32]	-	-	no association	no association
	Naghshi et al. [100]	increased association	no association	no association	no association
	Schwingshackl et al. [43]	-	-	no association	no association
**CV mortality**	Mazidi et al. [97]	-	-	no association	no association
	Naghshi et al. [100]	increased association^d^	no association	no association	no association
**Others**					
**T2DM**	Chen et al. [105]	-	-	no associaton	no association
	Feng et al. [41]	-	-	no association	no association
	Gijsbers et al. [106]	-	-	no association	no association
	Mohan et al. [103]	no association	no association	no association	no association
	Schwingshackl et al. [43]	-	-	no association	inverse association

“-“ indicates that no included papers reported analyses on these associations.

^a^Systolic blood pressure.

^b^(pre-menopausal population only), no association post-menopausal.

^c^Low-fat only; skim milk, no association.

^d^Only in high vs. low analysis, not in linear dose–response analysis.

^e^Only RCTs included, thus measure of effect.

## Results

**Fig. 1:**
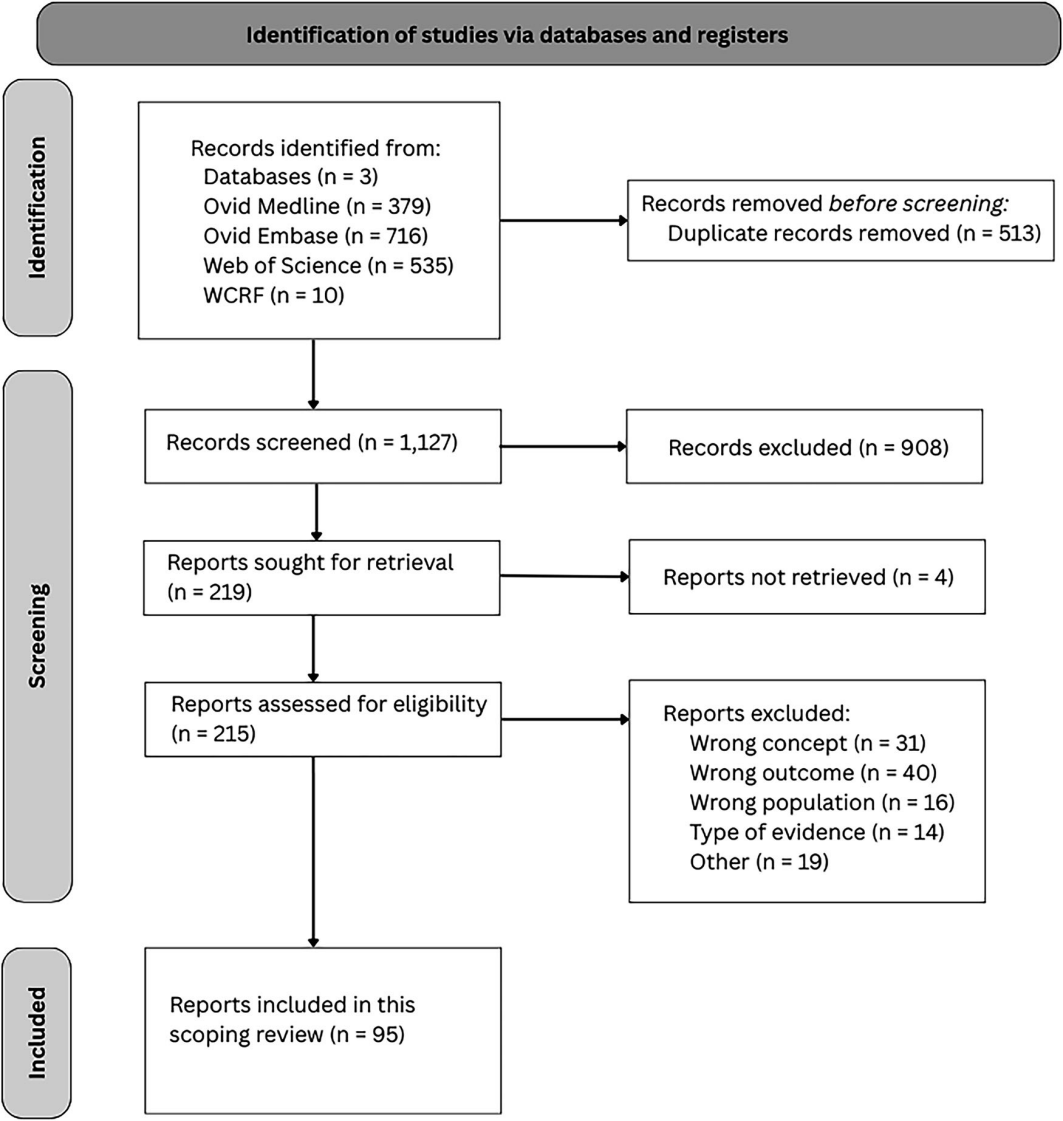
Flow diagram of the study selection.

The literature search yielded 1630 results: 379 from MEDLINE, 716 from EMBASE, and 535 from Web of Science. In addition, ten WCRF evidence synthesis reports, which can be categorized as systematic reviews, were included [22]. Two hundred and fifteen records were assessed as full texts, and a total of 95 reports were included in this scoping review, of which 92 contained meta-analyses. The title, abstract, and full-text phases, along with reasons for exclusion, are presented in a PRISMA flowchart (Fig. 1) [23] A list of excluded reports with reasons for exclusion can be found in Supplemental Table C.

### Characteristics of included reports

The largest portion of included reports incorporated more than one study design (*n* = 43), followed by reports that included solely prospective cohort studies (*n* = 35). Seven reports examined follow-up data from RCTs, two were based exclusively on RCTs and four only included case–control studies (Supplementary Table A). Ten SRs were from the WCRF’s Continuous Update Project and include cohort studies, case–control studies and RCTs. The number of studies analyzed in the included reports ranged from five [24] to 152 [25], whereas participant numbers varied from 1427 [26] to 7,606,009 [27]. WCRF reports did not consistently provide the number of participants per included study in the report body, particularly for individual exposures/interventions such as dairy.

### Health outcomes

Twenty-nine diet-related health outcomes were identified in the included reports. They fell into five groups:

I. **cardiovascular outcomes** including composite cardiovascular disease [27–34], coronary heart disease [9, 29–33, 35–39], all types of stroke [9, 29–31, 33–37, 39, 40], ischemic stroke [38], and hypertension [9, 17, 41–43],
II. **cancer types** including bladder [28, 44–48], breast [49–56], colorectal [28, 52, 57–64], corpus uteri [65, 66], esophagus [28, 67, 68], kidney [69], leukemia [70], liver [71–73], lung [74, 75], non-Hodgkin lymphoma [76, 77], oral [78–80], ovarian [81–83], pancreatic [24], prostate [52, 84, 85], stomach/gastric [86–90], and “all types” [28],
III. **body composition** including overweight and obesity [41, 91], and weight gain [25, 91–93],
IV. **mortality** including all-cause mortality [32, 35, 94–100] and cardiovascular mortality [94–96, 100, 101],
V. **other outcomes** including T2DM [34, 41, 102–107], bone health [108–111], joint health including rheumatoid arthritis and knee osteoarthritis [112, 113], and cognitive outcomes [114–116].

**Fig. 2:**
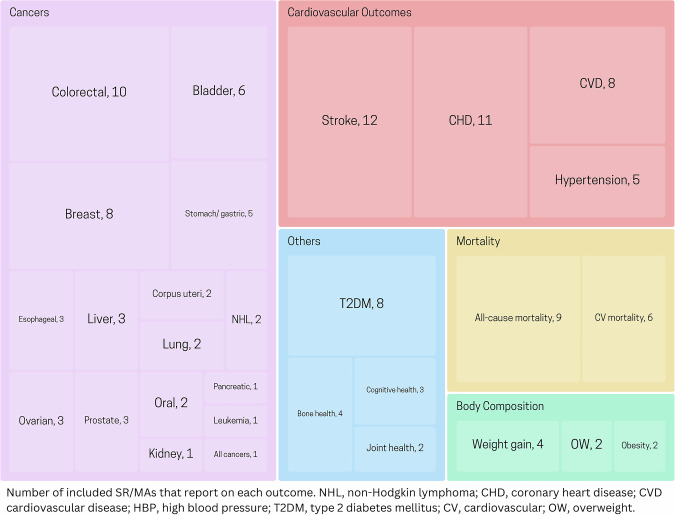
Treemap of included health outcomes.

Health outcomes were represented with varied frequency across the reports as illustrated in Fig. 2. The total number of associations exceeds the number of included reports, as several reports addressed multiple health outcomes. Supplementary Table D provides a tabular summary.

### Exposures and interventions

Exposures addressed in the included reports encompassed a range of dairy foods. In our analysis, we focused on total dairy, milk, yogurt, cheese, and fermented dairy products. These categories were determined by the authors of the included reports and may represent varying definitions. Broadly, “milk” refers to all milk consumption (not by sub-type), “total dairy” refers to all dairy products included in the individual reports, and “fermented dairy” refers to any fermented dairy foods categorized in individual reports as “fermented dairy”. Sixty-six reports assessed the association of total dairy on health outcomes, 52 examined associations with milk, 43 reports studied cheese and 40 investigated yogurt consumption. Thirteen reports presented findings about all fermented dairy products on health outcomes. A detailed overview can be found in Supplementary Table E.

### Synthesis of results

Table 1 provides a comprehensive overview of the health outcomes associated with the consumption of five dairy product categories: total dairy, milk, cheese, yogurt, and fermented dairy products. Detailed information on how each report was charted is provided in Supplemental Table E.

Out of the total 281 identified associations, 106 (37.7%) suggested that dairy consumption is associated with a reduced risk of certain negative health outcomes. Meanwhile, 135 (48.0%) indicated a neutral effect of dairy on the investigated outcomes. Twelve associations (4.3%) found that dairy consumption may increase the risk of certain negative health outcomes, and 28 (10.0%) yielded inconclusive results.

In the following sections, we will examine the associations of different dairy exposures—total dairy consumption, milk, cheese, yogurt, and overall fermented dairy—on the five outcome categories listed in Table 1: cardiovascular outcomes, individual cancer types, body composition, mortality, and other outcomes. We highlight the most significant findings for each exposure category.

#### Overall dairy intake

Sixty-six reviews examined associations between overall dairy consumption and health outcomes. This is not an aggregate measure; we are reporting findings of included reports that presented associations with overall dairy consumption. Our findings show that dairy intake is either associated with reduced risk (*n* = 35) or has no association (*n* = 40) with most of the health outcomes considered. Six associations yielded inconclusive findings, and five showed that overall dairy consumption was linked to an increased risk of negative health outcomes (Table 1). Overall dairy intake was associated with a reduced or neutral risk of all of the cardiovascular outcomes listed in Table 1 [9, 26, 29, 31–34, 36–38, 40–43, 86,101] except cardiovascular mortality. A lower risk of bladder [44], breast [53–55], colorectal [57, 61–64], and oral cancers [79], as well as overweight/obesity [41, 93], T2DM [34, 41, 102, 103, 106, 107], and joint health [113] was associated with overall dairy consumption. Some reports found that increased risk of liver cancer [73], non-Hodgkin lymphoma [77], ovarian [81], prostate [85], and gastric [89] cancers were associated with overall dairy consumption.

#### Milk intake

Fifty-one of the included reports investigated the association of milk intake with health outcomes. Of these, thirteen associations indicated a decreased risk, 43 were neutral, four suggested an increased risk, and ten yielded inconclusive findings on the identified health outcomes (Table 1).

The results indicated that milk consumption was associated with either a decreased risk (*n* = 4) or neutral associations (*n* = 11) on cardiovascular outcomes, whereas two inconclusive findings were reported [9, 29, 32, 35, 36, 38–42]. Overall, milk consumption was associated with either a reduced risk or no risk of developing most cancers. Specifically, reports suggest that milk consumption is associated with a lower risk of the development of oral [78, 79], bladder [44, 47], and colorectal cancers [57, 62]. Four reports suggested that milk was linked to an increased risk of certain types of cancer, including one report each for breast [50], prostate [85], liver cancer [71], and non-Hodgkin lymphoma [76]. Only one report explored the relationship between body composition and milk intake [41], and it found a reduced risk of overweight and obesity.

Associations between milk intake and cardiovascular- and all-cause mortality were heterogeneous: five neutral and two inconclusive associations were reported [32, 35, 94, 97, 100]. One report showed that consuming milk was linked to a reduced risk of developing T2DM [107], while three reports identified neutral associations [41, 103, 106]. Neutral (*n* = 1) or inconclusive (*n* = 8) associations were found between milk consumption and bone health, while four inconclusive and two neutral associations were found for cognitive outcomes [114–116]. No reports examined the relationship between milk consumption and leukemia, “all cancers,” joint health, or weight gain.

#### Cheese intake

Forty-two reports investigated associations between cheese intake and various health outcomes (Table 1). Overall, 20 associations showed a decreased risk, 25 had neutral findings, two showed an increased risk, and seven had inconclusive results across the health outcomes studied. Eight reports examined associations between cheese consumption and stroke [9,29–31,36,38–40] and seven investigated its relationship with CHD [29–31,38]. Some reports found that that cheese intake was associated with a lower incidence of cardiovascular diseases [29–32,36,38–40,117] while others reported neutral results [29, 32, 38, 39, 41, 42]. One report did not draw definitive conclusions [9]. Cheese was linked to a lower risk of developing cancer (breast, colorectal, oral, ovarian) in some reports [28, 50, 57, 60, 78, 81], but no associations were found in others (bladder, breast, colorectal, uterine, esophageal, liver, gastric, non-Hodgkin lymphoma) [24, 45, 46, 54, 58, 62, 63, 66, 67, 71, 72, 76, 86, 118]. An increased risk of prostate cancer was observed in one report [85]. Three reports found no association between cheese consumption and mortality [32, 97, 99], whereas relationships with bone health were inconsistent: one report showed positive associations and two were inconclusive [108, 110]. Similarly, two reports showed no link between cheese intake and T2DM [41, 103] while one found an increased risk of T2DM [105]. Only one report examined the association between cheese and cognitive outcomes [116], noting inconclusive findings. None of the reports investigated possible associations between cheese consumption and lung cancer, kidney cancer, leukemia, body composition, overweight, obesity, joint health, or cognitive disorders.

#### Yogurt intake

Forty reports investigated yogurt consumption. Overall, yogurt intake was found to have 25 associations with reduced risk, 25 neutral findings, no increased associations, and five inconclusive results with the identified health outcomes. One report found that yogurt intake was linked to a decreased risk of adverse cardiovascular outcomes [117], while seven found no associations with these outcomes [27, 29, 32, 36, 38, 41, 42]. Yogurt intake was associated with either a reduced risk [45, 49, 54, 57, 59, 67, 71, 72, 78, 117] or no association with cancer development in all reports [50, 51, 60, 73, 76, 82, 85, 118, 119] except one, which reported inconclusive evidence of the association between yogurt consumption and colorectal cancer [63]. There was also evidence that yogurt consumption is associated with a reduced risk of developing bladder [28, 45], breast [49, 54], colorectal [28, 57, 59], esophageal [67], liver [71, 72], and oral cancers [78]. Two reports investigated body composition outcomes. One found that yogurt intake reduced the risk of weight gain [92] while the other found no association with overweight or obesity [41]. Two reports found that yogurt consumption was associated with a decreased risk of cardiovascular- and all-cause mortality [95, 96] while another reported neutral findings on all-cause mortality [32]. Five reports indicated that yogurt intake was linked to a reduced risk of developing T2DM [41, 103, 106, 107, 120]. None of the included reports found that yogurt consumption was linked to an increased risk of any of the included 29 adverse health outcomes, though two reports were unable to draw definitive conclusions [9, 63].

#### Fermented dairy intake

Thirteen included reports investigated associations of overall fermented dairy intake, such as cheese, yogurt, and cultured/sour milk, on health outcomes. Overall, 13 associations showed a reduced risk of poor health outcomes, four found neutral results, and one indicated an increased risk. Fermented dairy was associated with a decreased risk of cardiovascular disease [32, 117] and stroke [36], whereas both a reduced risk [42], and no association [41] were seen with hypertension. Fermented dairy intake was also associated with a lower risk of developing bladder [28, 46], breast [55], and esophageal cancer [28], as well as T2DM [103, 120], and all-cause mortality [32]. No association between fermented dairy consumption and colorectal cancer [57, 58] were found in two reports, while another report demonstrated a decreased risk [28]. A single report found an association between the consumption of fermented dairy and risk of all-cause mortality [97]. No reports investigated the association of fermented dairy consumption with body composition.

#### High- versus low-fat dairy and milk

Twenty-seven of the included papers reported subanalyses of high- versus low-fat milk and dairy consumption (Table 2). Most of the included studies focused primarily on dairy products in aggregate rather than milk alone, and predominantly investigated cardiovascular outcomes [9, 26, 29, 31–34,36–38,40–43]. In addition to cardiovascular health, associations between dairy fat content and various other outcomes were reported. These included four cancer types—breast, colorectal, ovarian, and prostate cancer [51, 52, 54, 55, 57, 61, 63, 81, 82, 85]—as well as body composition [41], mortality [32, 97, 98, 100], and T2DM [41, 102, 103, 105, 106]. Across outcomes, most reviews reported no association between fat content of milk or dairy foods and adverse health outcomes. Inverse associations with both full-fat and reduced-fat dairy were reported for many cardiovascular outcomes [9, 26, 29, 31, 33, 36, 40–42], though two studies reported increased associations for full-fat milk [36, 38]. With reference to cancers, most reports found either no association or inverse associations between full-fat dairy consumption and adverse health outcomes. A notable exception was found in one report showing an increased association between full-fat milk and ovarian cancer, contrasting with an inverse association for reduced-fat milk [81]. While most reports found no association between low- or high-fat milk or dairy and mortality, one reported an increased association of full-fat milk with both all-cause mortality and cardiovascular mortality [100].

## Discussion

This scoping review aimed to map the existing research on dairy consumption and its associations with various health outcomes. Overall, nearly half of the reported associations (48.0%, *n* = 135) showed no significant link to adverse health effects. Our findings indicate that a substantial body of research supports the view that dairy intake is generally associated with neutral or positive associations across a range of clinical outcomes, particularly regarding common non-communicable diseases. This scoping review suggests that current scientific evidence contradicts the skepticism and negative messaging often found in the media regarding the health effects of dairy consumption [121–123], which has contributed to a growing trend of replacing dairy products with plant-based alternatives [122, 123]. Notably, dairy products have a markedly different nutritional profile than non-dairy products, offering higher levels of protein and key micronutrients [121, 124].

Our results indicate that dairy consumption is linked to either moderate benefits or neutral associations with cardiovascular and metabolic health outcomes. This has broad public health implications, especially considering the high prevalence of obesity and the fact that cardiovascular disease is the leading cause of disability and premature death in high-income countries, and the second leading cause in middle- and low-income countries [125, 126]. Specifically, dairy intake is not linked to overweight or obesity, which is particularly relevant given the global epidemic. Additionally, dairy intake is not associated with an increased risk of T2DM, a major comorbidity of obesity, and fermented dairy products may even reduce the risk of T2DM, although the underlying mechanisms remain unclear [41, 103, 104, 106, 107].

Overall, we found that dairy intake was associated with a reduced risk of colorectal cancer, which aligns with a recent large prospective cohort study [127]. This is a particularly notable finding, as colorectal cancer is the third most common cancer worldwide [128]. Given its high incidence, even modest reductions in colorectal cancer risk associated with dietary factors like dairy intake could have meaningful implications for cancer prevention strategies.

Our findings are consistent with the results of a recently-published umbrella review by Zhang et al., who investigated the effects of milk consumption on various health outcomes in children and adults [129]. The report included 41 meta-analyses covering 45 health outcomes. The primary conclusion was that milk intake was more often associated with beneficial health effects than with harmful ones. Specifically, the analysis revealed that milk intake was linked to a reduced risk of cardiovascular disease, stroke, hypertension, colorectal cancer, obesity, T2DM, Alzheimer’s disease, metabolic syndrome, and osteoporosis. In contrast, the authors also found associations between milk and an increased risk of developing prostate cancer and Parkinson’s disease. While Zhang and colleagues focused exclusively on milk consumption, our study expands their analysis by including a broader range of dairy products, while narrowing the scope of health outcomes and offering a more detailed review.

When examining different dairy product categories, we observed significant variability in the direction of associations across the types of dairy products. These differences may be partly attributed to variations in methodology and study design among the included studies. Additionally, the nutrient profiles of individual dairy products themselves can vary greatly and may influence their potential health impacts. Notably, the categories yogurt and fermented dairy showed the highest percentages of associations with reduced disease risk. This suggests that these products may offer unique health benefits compared to other dairy foods. Fermented dairy is rich in probiotics and other bioactive compounds that may have anti-inflammatory properties, potentially benefiting cardiovascular, metabolic, and gut health [11]. However, the categories were examined in fewer reports than those focusing on milk and cheese: 40 reports on yogurt, and 13 on all fermented dairy, compared to 65 on total dairy, 51 on milk, and 42 on cheese consumption. Future studies on fermented dairy products are needed to better understand their specific health benefits.

Findings on cheese intake and health outcomes were more heterogeneous, predominantly indicating positive or neutral associations. Although some cheese varieties contain high amounts of saturated fat and sodium, observational studies generally do not support the idea that cheese consumption increases the risk of CVD [130]. One possible explanation is that the fatty acid composition of cheese differs from that of other dairy products. For instance, odd-chain saturated fatty acids, such as C15:0 and C17:0, may be present in higher concentrations in cheese than other dairy foods, and is associated with a lower risk of cardiovascular diseases and T2DM in observational studies [131]. Moreover, consuming cheese is associated with a reduction in LDL-cholesterol, which is a well-established biomarker for cardiovascular diseases [132–134].

Some studies examining prostate cancer found an increased risk associated with all dairy, milk, and cheese consumption (Table 1). Though a clear biological explanation has not been confirmed, it has been suggested that higher intake of dairy products are linked to higher circulating concentrations of insulin-like growth factor 1, inflammatory biomarkers, and estrogen, which may promote the proliferation of prostate cancer cells [85, 135]. Notably, this evidence is limited and other risk factors such as age, ethnicity, and family history are stronger determinants of prostate cancer risk [135]. Therefore, from a public health perspective, the scientific evidence is not robust enough to recommend avoiding dairy intake for prostate cancer prevention [84].

It is surprising that only four reports on bone health met our inclusion criteria. After evaluating the available literature, we found that the most studies in adults focus on calcium rather than on dairy per se, with much of the dairy-related research centered on pediatric populations [136]. Studies in adults often investigate dairy in the form of milk powder or calcium supplements [137, 138], which did not meet the inclusion criteria for this review.

### Strengths and weaknesses of the study

This scoping review integrates systematic reviews and meta-analyses of both observational studies and RCTs, offering a comprehensive overview of the available evidence on dairy intake and chronic disease outcomes. Each study type provides unique strengths that contribute to the overall understanding of this topic. Observational studies, including cohort and case–control designs, are particularly valuable for identifying long-term associations. Prospective cohort studies, which collect exposure data before outcomes occur, are effective in minimizing recall bias and exploring temporal relationships between dairy and the disease outcome. In contrast, RCTs have more control over confounding variables and are ideal for investigating causal pathways and disease risk markers. However, each study design also has limitations that must be considered. While cohort studies are critical for understanding long-term effects, they are susceptible to confounding factors such as changes in health status, behavior, or aging, and may suffer from regression dilution [139], particularly if dairy intake was only assessed at baseline. Case–control studies, being retrospective, are more prone to recall and selection biases, which can affect the reliability of their findings. RCTs are often shorter in duration and may not fully capture the long-term outcomes that are crucial for understanding chronic diseases, though the majority of the included RCTs investigated follow-up data. The respective strengths and weaknesses of each of these study types highlight the need to carefully consider the quality of evidence from each design when interpreting the broader patterns and gaps in the literature.

The breadth of scoping reviews can introduce additional limitations. One challenge is presenting data from subgroup analyses conducted within individual reports. Similarly, reporting nonlinear associations is difficult in a review of this scale. Some reports lacked clarity in their findings, while others presented mixed results depending on analysis type, for example high/low intake and dose-response analysis [38, 41, 85, 108]. Due to the variation in analytical methods and the complexity of within-report findings, extracting and reporting results from some papers was challenging. As a result, the findings from some reports were categorized as inconclusive. Additionally, including systematic reviews carries the risk of overlapping data, as the same study populations may appear in multiple reports, potentially inflating associations for certain outcomes.

Only 36 (48%) of the reports that included both men and women stratified results by sex (Supplementary Table A). This represents a significant limitation in many studies, as failing to account for sex differences can obscure important variations in health outcomes. Future research should prioritize stratifying results by sex to better understand these differences and provide more tailored and effective public health recommendations.

A limitation in studies on dairy consumption is the lack of standardization in the products being compared. Dairy products vary widely in composition, depending on factors such as country of origin, brand, and the specific animal environments in which the dairy is produced. These variations can lead to inconsistencies that are difficult to control for when comparing study results. For example, several countries have implemented mandatory or voluntary vitamin D fortification policies for dairy products in response to widespread deficiencies [140]. As a result, dairy products from different regions or brands may have differing nutrient profiles, which can influence health outcomes in ways that are not captured in many studies. The reports we included did not provide information on whether the dairy consumed by participants was fortified with vitamin D. As a result, it is difficult to draw definitive conclusions from these reports about the role of vitamin D in milk and other dairy products and how it might affect health outcomes.To address this, future research should aim to differentiate between the effects of fortified and non-fortified dairy consumption, as well as consider other variations in dairy products such as fat content and processing methods.

## Conclusions

In summary, this review highlights that dairy consumption is generally linked with neutral or protective associations with adverse health outcomes. Certain dairy foods, particularly yogurt and fermented dairy, consistently showed evidence of health benefits. However, variability in findings, especially regarding cheese intake, underscores the need for further investigation.

To strengthen the evidence base and reduce regression dilution, future research in prospective cohorts should prioritize more frequent dietary assessments, such as annual food frequency questionnaires, to better capture changes in dietary habits, as studies show that dairy product consumption can fluctuate considerably over 4-year periods [141]. Ultimately, enhancing the methodological rigor in assessing diet over time will provide deeper insights into the complex relationship between dairy intake and chronic disease, facilitating more effective prevention and intervention strategies.

## Supplementary information

Supplementary Tables for: Association between dairy intake and multiple health outcomes: a scoping review of systematic reviews and meta-analyses.

## Data Availability

All data presented in the text are available in the included tables and supplemental files. Data are available upon reasonable request to the corresponding author.
