# Supplementary material for: Association between dairy intake and multiple health outcomes: a scoping review of systematic reviews and meta-analyses
Source: Eur J Clin Nutr. 2025 Jul 26;80(1):16–27. doi: 10.1038/s41430-025-01639-5 (PMC12783052; doi:10.1038/s41430-025-01639-5)
Supplement: Supplementary file 1 — Supplementary Tables for: Association between dairy intake and multiple health outcomes: a scoping review of systematic reviews and meta-analyses. [file 41430_2025_1639_MOESM1_ESM.pdf]

Supplemental Tables for:

Association between Dairy Intake and Multiple Health Outcomes: A Scoping Review of Systematic Reviews and Meta-Analyses

Authors: Saskia Akyil[1*] and Stefanie Winkler[1*], Dorothy Meyer[1], Eva Kiesswetter[2], Martin Kussmann[3,4], Lukas Schwingshackl[2], Hans Hauner[1]

Affiliations:

[1] Institute of Nutritional Medicine, Else Kröner-Fresenius-Center for Nutritional Medicine, TUM School of Medicine and Health, Technical University of Munich, Munich, Germany

[2] Institute for Evidence in Medicine, Medical Center - University of Freiburg, Faculty of Medicine, University of Freiburg, Freiburg, Germany

[3] Competence Center of Nutrition (KErn) at the Bavarian State Research Center for Agriculture, Freising, Germany

[4] Kussmann Biotech GmbH, Nordkirchen, Germany

* These authors contributed equally to this work.

Corresponding author:

Hans Hauner

Institute of Nutritional Medicine, Else Kröner-Fresenius-Centre for Nutritional Medicine, TUM School of Medicine and Health, Technical University of Munich, Georg-Brauchle-Ring 62, 80992 Munich, Germany Telephone: +49 (0) 89 289249-21, email: hans.hauner@tum.de

Supplemental Files:

Supplemental Table A: Extraction table of included papers

Supplemental Table B: Extraction table of World Cancer Research Fund included papers

Supplemental Table C: Excluded papers

Supplemental Table D: Papers charted by health outcome

Supplemental Table E: Papers charted by exposure

Supplemental Table F: Charting of author-assigned paper IDs in Table 1

Supplemental Table G: Ovid Medline Search Strategy

Supplemental Table H: Ovid Embase Search Strategy

Supplemental Table I: Web of Science Search Strategy

Supplemental Table A: Extraction table of included papers

Title, author, year	ID	Paper type: systematic review (SR), SR with MA: SR/MA	Outcome	Aim of review	Study designs of included studies	Number of Studies included	Participant Count	Region	Interven-tion/ exposure included in this review	Compar-ison	Outcome measured	Evidence: protective, neutral, harmful, or conflicting	Date range of included studies	Strati-fied by sex?
A systematic review and meta-analysis of observational studies on the association between animal protein sources and risk of rheumatoid arthritis (Asoudeh, 2021)	1	SR/MA	rheumatoid arthritis	The aim of the review is to investigate the linear and nonlinear dose-response associations of animal-based dietary protein intake and the risk of developing rheumatoid arthritis (RA).	Prospective Cohort Studies & Case-control studies	13 total Including dairy as exposure: 7 cohort studies 6 case-control studies	466,800	Sweden, USA, Denmark Greece, France, China, Iran	dairy	high/low intake dose response	Risk of developing rheumatoid arthritis (RA)	neutral	up to October 2020.	No
Association between Dairy Intake and Gastric Cancer: A Meta-Analysis of Observational Studies (Tian, 2014)	2	SR/MA	Cancer, Gastric cancer	The aim of the review was to summarize the evidence on the relationship between dairy intake and gastric cancer risk, investigating associations based on different study designs, geographic areas, gastric cancer subtypes, and gender.	18 case-control and 8 cohort studies	26 studies	230,627	Asia, Europe, North America, South America	dairy	High versus low dairy product intake<br><br>fermented vs. no fermented dairy consumpti on	The risk of gastric cancer	Cheese and gastric cancer: neutral milk and gastric cancer, neutral	up to August 2013	No
Association between dairy intake and the risk of contracting type 2 diabetes and cardiovascular diseases: a systematic review and meta-analysis with subgroup analysis of men versus women (Mishali, 2019)	3	SR/MA	Type 2 Diabetes, Cardiovas-cular Diseases	The aim of the review is to investigate the effects of dairy consumption on the risk of developing type 2 diabetes (T2D) and cardiovascular disease (CVD) in men and women.	30 Prospective Cohort studies	30 total Number of T2DM Studies: 16 Number of CVD Studies: 14	1,006,475	UK, Sweden, Australia, Japan, USA, Europe, Denmark, China	dairy	high versus low dairy intake<br><br>men vs. women	Risk of developing type 2 diabetes (T2D) and cardiovascul ar disease (CVD)	dairy: protective T2D dairy: protective CVC, stroke	2006 – Nov. 2016	yes

Association Between Dairy Product Consumption and Colorectal Cancer Risk in Adults: A Systematic Review and Meta-Analysis of Epidemiologic Studies (Barrubés, 2019)	4	SR/MA	Cancer, Colorectal cancer	The aim of this systematic review and meta-analysis was to extend the available evidence and combine all the results from prospective cohorts and case-control studies in adults so that the association between the consumption of specific types of dairy products and CRC incidence could be examined.	15 Prospective Cohort Studies 14 Case-control Studies	29 total: - cohort studies: 15 - case-control studies: 14	1,396,167	US, Norway, Sweden, China, Italy, Spain, Europe, Nether-lands, France, Japan, Canada, Australia, Korea	total dairy products, total milk, whole milk, fermented dairy products, total yogurt, cultured milk or cheese	high/low intake	Risk of developing colorectal cancer (CRC)	total dairy: protective<br><br>milk: protective<br><br>yogurt: protective<br><br>cheese: decreased risk<br><br>fermented dairy: neutral	up to June 4, 2018	No
Cheese Consumption and Risk of All-Cause Mortality: A Meta-Analysis of Prospective Studies (Tong, 2017)	5	SR/MA	Mortality	The aim of the review was to investigate the association between cheese consumption and the risk of all-cause mortality	Prospective studies	9	177,655	The Netherlands, UK, United States, Italy, Australia	cheese	High vs. low cheese consumption, dose-response relationship	Risk of all-cause mortality	neutral	January 1997 to December 2015	Yes
Cheese consumption and risk of cardiovascular disease: a meta-analysis of prospective studies (Chen, 2017)	6	SR/MA	cardiovascular disease	The aim of the review is to investigate the association between cheese consumption and the risk of cardiovascular disease (CVD), specifically looking at the risks of total CVD, coronary heart disease (CHD), and stroke.	Prospective Cohort Studies.	15	Not available	Europe, USA, Australia	cheese	high/low intake, dose/response	Risk of cardiovascular disease (CVD), including total CVD, coronary heart disease (CHD), and stroke	Protective: CVD, CHD, stroke	PubMed (Jan 1, 1966–Dec 15, 2015) EMBASE (Jan 1, 1980–Dec 30, 2015)	Yes
Colorectal cancer and non-fermented milk, solid cheese, and fermented milk consumption: A systematic review and meta-analysis of prospective studies (Ralston, 2014)	40	SR/MA	Cancer - colorectal, colon, rectal	To examine the association between intake of different types of dairy foods during adulthood and the dvmt of CRC, specifically colon cancer & rectal cancer separately in men & women, and to compare the effects of non-fermented fluid milk, solid cheeses, and fermented milks.	Prospective Cohort Studies	15	Total: 919,680	Norway, Finland, USAS, France, Sweden, China, UK	dairy: non-fermented milk, solid cheese, and fermented milk	Low/high intake of different types of dairy	colorectal cancer (CRC), colon cancer, and rectal cancer	neutral:solid cheese, fer-mented milk: CRC, colon, rectal cancers neutral:non-fermented milk: rectal cancer men; colon or rectalcancer women protective: non-fermented milk: colon cancer men	January 1990 - July 2009	Yes

Comparative effectiveness of single foods and food groups on body weight: a systematic review and network meta-analysis of 152 randomized controlled trials (Jayedi 2023)	41	SR/MA	Body Composition - body weight	The aim of the review was to quantify and rank the effects of different single foods or food groups on weight loss in adults.	RCTs with an intervention duration of 4 weeks or longer	152	Total: 9669	Worldwide - all continents	dairy	One of food groups or usual diet/no intervention	Weight loss in kilograms (kg), health-related quality of life (HRQoL), adverse events	Neutral: dairy on weight loss	January 1983 to December 2020	No
Consumption of dairy foods and diabetes incidence: a dose-response meta-analysis of observational studies (Gijsbers, 2016)	7	SR/MA	Type 2 Diabetes	To investigate the associations between incident type 2 diabetes (T2D) and different levels of intake of dairy foods, including total dairy, low-fat dairy, yogurt, ice cream, and other dairy types, focusing on dose-response relationships.	Prospective Cohort Studies.	22	579,832	United States, Europe, Asia, and Australia	total dairy, yogurt, milk	1. dose/response for total dairy, low-fat dairy, and high-fat dairy; 2. intake amounts/ no intake for yogurt and ice cream; 3. consumed/not consumed for other dairy types not associated with T2D risk.	Incidence of type 2 diabetes (T2D) in relation to dairy food consumption	protective: total dairy, yogurt neutral: milk	Inception to April 2015.	Yes

Consumption of dairy product and its association with total and cause specific mortality e A population-based cohort study and meta-analysis (Mazidi, 2019)	42	SR/MA	Mortality, cerebrovascular mortality, CHD mortality	The aim of the review was to investigate the associations between dairy product consumption and total and cause-specific mortality through a systematic review and meta-analysis of existing prospective cohort studies among American adults.	Prospective Cohort Studies	12	636,726	North America	dairy, cheese milk, yogurt	dairy intake, milk intake, fermented dairy intake, cheese intake	total mortality, cerebrovascular mortality, CHD mortality	neutral: total dairy and CHD mortality neutral: total dairy and cancer mortality neutral: milk and total mortality harmful: milk and CHD mortality harmful: fermented dairy and total mortality	January 2007 to December 2017	Yes
Consumption of Dairy Products and the Risk of Overweight or Obesity, Hypertension, and Type 2 Diabetes Mellitus: A Dose-Response Meta-Analysis and Systematic Review of Cohort Studies (Feng, 2022)	8	SR/MA	Overweight or Obesity, Hypertension and Type 2 Diabetes Mellitus	The aim of the review was to investigate the associations between different types of dairy product consumption (total dairy, low-fat dairy, high-fat dairy, fermented dairy, milk, yogurt, and cheese) and the risk of overweight or obesity, hypertension, and type 2 diabetes mellitus (T2DM).	Prospective Cohort Studies	42	1,212,693	Asia, Europe, Australia, United States, multinational	total dairy, milk, yogurt, cheese	Obesity/ overweight: dose-response associations between different types of dairy products<br><br>Hypertension: dose-response associations between different types of dairy products<br><br>T2DM: Dairy product consumption	Risk of overweight or obesity, hypertension, and type 2 diabetes mellitus.	T2D: protective: total dairy, yogurt neutral: cheese, milk<br><br>Overweight: protective: milk, total dairy neutral: yogurt<br><br>Hypertension: protective: total dairy, milk neutral: fermented dairy, yogurt, cheese	Up to April 22, 2021	Yes
Consumption of milk and dairy products and risk of osteoporosis and hip fracture: a systematic review and Meta-analysis (Malmir, 2020)	9	SR/MA	Osteoporosis and hip fractures	The aim of the review was to summarize earlier data on the association between milk and dairy intake and the risk of osteoporosis and hip fracture through a meta-analysis.	Cross-sectional Studies, Case-control Studies, Prospective Cohort Studies	15	616,420	Europe, North America, Asia	- milk<br>- dairy<br>- cheese<br>- yogurt	Amount of consumption	risk of osteoporosis and hip fracture	conflicting: milk, all dairy	published between 1993 and 2017	yes

Consumption of Yogurt and the Incident Risk of Cardiovascular Disease: A Meta-Analysis of Nine Cohort Studies (Wu, 2017)	10	SR/MA	cardiovascular disease	The aim of the review was to investigate the association between yogurt intake and the incident risk of cardiovascular disease (CVD) specifically. The review aimed to provide evidence on whether yogurt consumption, especially at a dose of ≥200 g/day, could be associated with a lower incident risk of CVD.	prospective cohort studies	9	291,236	North America, Europe	yogurt	Highest category vs. lowest category of yogurt consumption, ≥200 g/day yogurt intake vs. <200 g/day yogurt intake, dose response	Yogurt Consumption and the Occurrence of CVD	neutral	Publication range between 1999 and 2015	Yes
Daily milk consumption and all-cause mortality, coronary heart disease and stroke: a systematic review and meta-analysis of observational cohort studies (Mullie, 2015)	11	SR/MA	Mortality, coronary heart disease, stroke	The aim of the review was to investigate the associations between daily milk consumption and all-cause mortality, coronary heart disease, and stroke through a systematic review and meta-analysis of observational cohort studies.	Prospective Cohort Studies	21	603,066	multinational: United States, Asia, Europe, Australia	milk	Different levels of milk consumption (200 mL/d)	All-cause mortality, Coronary heart disease, Stroke	neutral	1984 to June 2015	Yes
Dairy consumption and CVD: a systematic review and meta-analysis (Alexander, 2016)	12	SR/MA	Cardiovascular disease	The associations investigated were total dairy intake and CVD, CHD, and stroke; individual dairy products (milk, cheese, yogurt) and CVD, CHD, and stroke; and calcium from dairy sources and CVD, CHD, and stroke.	Prospective Cohort Studies	31	over one million	USA, Europe, Nordic countries, Australia, Japan	total dairy intake, specific dairy products (e.g. milk, cheese, yogurt)	high/low intake	Total CVD, Total CHD, Total stroke	milk: neutral CHD, stroke<br><br>cheese: neutral CVD, protective: CHD, stroke<br><br>yogurt: neutral CHD, CVD<br><br>total dairy: neutral: CVD, CHD, protective: stroke	March 1996 to March 2015	Yes

Dairy Consumption and Gastric Cancer Risk: A Meta-Analysis of Epidemiological Studies (Guo, 2015)	13	SR/MA	Cancer, gastric cancer	The aim of the review was to review and summarize the epidemiologic evidence on the relation of dairy consumption with risk of gastric cancer and to examine the dairy consumption and gastric cancer association according to study characteristics.	case-control and cohort studies	23	627,654	Europe, United States, South America	- dairy<br>- milk<br>- cheese<br>- yogurt<br>- butter<br>- other (include types: cottage and other dairy products mentioned as part of the total dairy food definition)	highest versus lowest total dairy intake<br>- highest versus lowest milk intake	gastric cancer	dairy: conflicting results<br>milk: neutral	Publication range of included studies: 1974 - 2013	No
Dairy consumption and liver cancer risk: A meta-analysis of observational studies (Dai, 2024)	43	SR/MA	Cancer - liver	The present study performed a comprehensive meta-analysis to systematically evaluate the association between the risk of developing PLC and the consumption of dairy products, including milk, yogurt, cheese and curd.	10 cohort studies<br>8 case-control studies	18 studies	6,562,714	Europe, Asia, USA	Milk, yogurt, cheese, curd, total dairy	highest vs lowest consumption	Liver cancer	harmful: milk<br>protective: yogurt<br>neutral: total dairy, cheese	Until Dec 2022	No
Dairy Consumption and Liver Cancer Risk: A Systematic Review and Dose-Response Meta-Analysis of Observational Studies (Zhao, 2021)	14	SR/MA	cancer, liver cancer	The aim of the review was to investigate the associations between dairy product consumption (including total dairy, milk, cheese, curd, and yogurt) and the risk of liver cancer through a systematic review and meta-analysis.	cohort study, case control study	15	5121 cases. No total given	USA, Europe, Asia	1. dairy<br>2. milk<br>3. cheese<br>4. yogurt	Milk intake, total dairy intake, yogurt consumption, cheese and curd intake, and a 40 g/day increment of yogurt intake. dose response	Risk of liver cancer	milk, total dairy, cheese: neutral<br>yogurt: protective	January 1988 to December 2019	No

Dairy consumption and lung cancer risk: a meta-analysis of prospective cohort studies (Yu, 2016)	15	SR/MA	cancer, lung cancer	The aim of the review was to investigate the relationship between dairy consumption and lung cancer risk, as well as to assess the associations between different types of dairy products (such as milk and total dairy products) and lung cancer risk.	cohort studies	8	61,901	Scandinavia, Sweden, USA, Poland, UK, Iran	Milk (whole/high-fat milk and skim/low-fat milk) total dairy products	high vs low consumption, dose-response	lung cancer	dairy: neutal	January 1996 to December 2014	No
Dairy consumption and risk of cardiovascular disease: an updated meta-analysis of prospective cohort studies (Qin, 2015)	16	SR/MA	cardiovascular disease	The aim of the review was to investigate the association between dairy consumption and the risk of cardiovascular disease (CVD), stroke, and coronary heart disease (CHD) through a meta-analysis of prospective cohort studies.	Prospective Cohort Studies	22	849,120 participants	Europe, USA, Japan, Australia, Taiwan	dairy, yogurt, cheese and butter	Comparison: Consumption of yogurt & cheese in relation to the risks of stroke and CHD	Overall risk of CVD, Risk of stroke, Risk of CHD	dairy: significantly decreased CVD risk, significantly decreased risk of stroke, neutral for CHD cheese: decreased risk for CHD and stroke	1997 - 2013	No
Dairy consumption and risk of esophageal squamous cell carcinoma: A meta-analysis of observational studies (Li, 2016)	18	SR/MA	cancer, esophageal squamous cell carcinoma	The aim of the review was to investigate the relationship between consumption of dairy products and the risk of esophageal squamous cell carcinoma (ESCC). The associations investigated were between intakes of total dairy products, milk, cheese, butter, and yogurt and the risk of ESCC.	- 17 Case-control studies<br>- 2 Cohort studies	19	4,315	United States, South America, Europe, Asia	- dairy<br>- milk<br>- cheese<br>- butter<br>- yogurt	-high versus low dairy consumption<br>-dose-response analysis of total dairy product consumption.	Risk of esophageal squamous cell carcinoma (ESCC)	milk, dairy, cheese: neutral<br>yogurt: protective	up to January 31, 2013.	No
Dairy Consumption and Risk of Stroke: A Systematic Review and Updated Dose-Response Meta-Analysis of Prospective Cohort Studies (De Goede, 2016)	19	SR/MA	stroke	To investigate the associations between dairy consumption and stroke risk, focusing on milk, cheese, yogurt, butter, and total dairy, while considering factors such as continent, type of stroke, and fat content.	Prospective Cohort Studies	18	762,414	Europe, East Asia (China and Japan), United States, Australia	milk, Cheese, Yogurt, and Total Fermented Dairy, Total Dairy, and Butter	dose/response	Stroke risk.	total dairy: neutral<br>yogurt: neutral<br>total fermented dairy: reduced risk<br>cheese: reduced risk<br>milk: reduced risk	October 1989 to October 2015	Yes

Dairy consumption and risk of type 2 diabetes: 3 cohorts of US adults and an updated meta-analysis (Chen, 2014)	20	SR/MA	type 2 diabetes	The aim of the review was to investigate the association between dairy consumption and the risk of type 2 diabetes, specifically looking at total dairy and individual types of dairy products, with a focus on yogurt.	Prospective Cohort Studies	14	459,790	United States	total dairy<br>yogurt<br>cheese<br>whole milk	high/low intake	Risk of type 2 diabetes (T2D) in relation to dairy consumption with a specific focus on yogurt intake	yogurt: reduced risk<br><br>cheese: increased risk<br><br>total dairy: neutral	January 1980 to December 2010	No
Dairy Consumption and Risks of Colorectal Cancer Incidence and Mortality: A Meta-analysis of Prospective Cohort Studies (Jin, 2020)	92	SR/MA	Colorectal Cancer Incidence and Mortality	The aim of the review was to investigate the associations between dairy consumption and colorectal cancer incidence and mortality through a meta-analysis of prospective cohort studies.	Prospective Cohort studies	31<br>.	2,780,995	Europe, United States, Asia	dairy<br>milk<br>cheese<br>fermented milk	Highest versus lowest categories of consumption<br><br>Dose-response	Risk of colorectal cancer incidence and mortality	cancer incidence<br>all dairy: protective<br>milk: protective<br>cheese: neutral<br>colorectal	up to July 2019.	Yes
Dairy foods and risk of stroke: A meta-analysis of prospective cohort studies (Hu, 2014)	21	SR/MA	stroke	The aim of the review was to summarize the evidence from prospective cohort studies regarding the association between dairy foods and the risk of stroke, assess the risk of stroke for the highest vs. lowest categories of dairy foods consumption, evaluate the dose-response relationship of dairy foods with the risk of stroke, and assess heterogeneity among studies and publication bias.	Prospective Cohort studies	15	764,635	Asia, Europe	dairy<br>milk<br>cheese<br>fermented milk	Total dairy vs. no/low dairy<br>-Fermented milk vs. no/low dairy<br>-Cheese vs. no/low dairy<br>-Non-fermented milk vs. no/low dairy<br>- Different levels of milk consump-tion<br>- H vs. L milk consump-tion<br>- Fermented vs. nonfermented milk	Risk of stroke<br>8 studies about dairy foods and stroke mortality,<br>7 focused on dairy foods and stroke incidence.	milk: neutral<br>total dairy: protective<br>cheese: protective	Inception to November, 2013	yes

Dairy Intake and Risk of Cognitive Decline and Dementia: A Systematic Review and Dose-Response Meta-Analysis of Prospective Studies (Villoz, 2024)	22	SR/MA	cognitive decline, dementia	The associations investigated in the systematic review were the relationship between dairy intake and cognitive decline or incident dementia, exploring potential differences by sex, age, region of origin, level of intake, and type of dairy products.	cohort studies	15	312,580	Europe, Asia, Australia, United States	dairy	-Quantity of dairy consump-tion (high/ low intake) -Frequency of dairy consump-tion (higher/ lower frequency) -Type of dairy product (milk/ cheese) -Geo region	Risk of cognitive decline or dementia in relation to dairy intake, with a focus on the quantity and frequency of consumption	all dairy: conflicting milk, cheese: conflicting	From inception to 11 July 2023	Yes
Dairy intake and the risk of pancreatic cancer : the Japan Collaborative Cohort Study (JACC Study) and meta-analysis of prospective cohort studies (Arafa, 2022)	23	SR/MA	cancer, pancreatic cancer	The aim of the review was to assess the associations between the intakes of dairy products and the risk of PAC.	Prospective Cohort Studies	5	59,774	North America, Europe, Oceania, Norway, Japan	Milk Cheese Yogurt	Different frequencies of dairy product intake (milk, cheese, and yogurt)	Incidence of pancreatic cancer (PAC)	milk, cheese, yogurt: neutral	Untill 31.3.2021	No
Dairy Product Consumption and Bladder Cancer Risk: A Meta-Analysis (Wu, 2020)	44	SR/MA	Cancer - bladder	The aim of the review was to reassess the association between dairy product consumption and bladder cancer risk based on currently available epidemiological studies.	- Cohort Studies: 6 - Case-Control Studies: 16	26	226,145	Worldwide	dairy products, including milk, cheese, yogurt	High vs. low intake values of dairy products, geographical region, NOS score, impact factor, and study design	Bladder cancer risk	protective: milk protective: dairy	Up to to March 2018	yes
Dairy Product Consumption and Cardiovascular Health: A Systematic Review and Meta-analysis of Prospective Cohort Studies (Chen, 2022)	24	SR/MA	Cardio-vascular health	To assess the associations between total and specific dairy consumption and the risk of hypertension (HTN), coronary heart disease (CHD), and stroke in a SR and MA of prospective cohort studies.	Prospective cohort studies	55	Not available	United States, Europe, Asia, Multicontinental	Total dairy High-fat dairy Low-fat dairy Milk Cheese Yogurt	high/low intake and dose/response	Risk of hypertension (HTN), coronary heart disease (CHD), and stroke	total dairy: protective against hypertension, stroke and CHD<br><br>cheese, milk, and yogurt: conflicting for all 3	Up to August 1, 2020	Yes

Dairy product consumption and gastric cancer risk: A meta-analysis (Sun, 2014)	25	SR/MA	cancer, gastric cancer	To investigate whether dairy product consumption is a risk factor for gastric cancer.	10 cohort studies<br>29 case-control studies	39	848,863	Worldwide	dairy	highest vs lowest dairy consump-tion categories	gastric cancer risk	dairy: neutral	October 1980 to Septemb er 2013	Yes
Dairy product consumption and risk of hip fracture: a systematic review and meta-analysis (Bian, 2018)	26	SR/MA	hip fracture	The primary aim of our meta-analysis was to examine and quantify the potential association of dairy product consumption with risk of hip fracture	- Cohort Studies<br>- Case-control Studies	18	381,987	USA, Europe, Asia, Australia, Canada	dairy	high/low intake, dose/resp onse analysis	Risk of hip fracture	cheese, total dairy, yogurt, milk: conflicting	from inception to April 17, 2017	Yes
Dairy Product Consumption and Risk of Non-Hodgkin Lymphoma: A Meta-Analysis (Wang, 2016)	27	SR/MA	non-Hodgkin lymphoma	The aim of the review is to investigate the association between dairy product consumption and the risk of non-Hodgkin lymphoma (NHL)	13 case-control studies<br>3 cohort studies	16	25,139	North America, Asia, Europe, Latin America	total dairy product, milk, butter, cheese, yogurt,	dose response comparing the highest vs. lowest categories of consumpti on for each type of dairy product.	Risk of non-Hodgkin lymphoma (NHL)	total dairy: harmful<br>milk: harmful<br><br>cheese: neutral<br><br>yogurt: neutral	up to October 2015.	No
Dairy products consumption and the risk of hypertension in adults: An updated systematic review and dose-response meta-analysis of prospective cohort studies (Heidari, 2021)	28	SR/MA	hypertension	The aim of the review was to summarize the relationship between dairy products consumption and the risk of hypertension (HTN) based on an updated systematic review and dose-response meta-analysis of prospective cohort studies.	Prospective Cohort studies	16	353,303	Europe, United States, Asia	dairy<br>milk<br>cheese<br>yogurt<br>(incl in fermented dairy products)<br>other (fermente d dairy products incl cheese & yogurt)	- Highest vs. lowest<br>- Dose-response analysis	risk of incident hypertension	total dairy: protective<br><br>milk: protective<br><br>fermented dairy: protective<br><br>yogurt: neutral<br><br>cheese: neutral	The publicati on range of included studies from 2002 to 2020.	Yes

Dairy Products Intake and Endometrial Cancer Risk: A Meta-Analysis of Observational Studies (Li, 2018)	30	SR/MA	cancer, endometrial cancer	The aim of the review was to conduct a meta-analysis to evaluate the relationship between dairy products intake and endometrial cancer risk.	- 16 Case-control studies<br>- 2 Prospective cohort studies	18	456,513	North America, Europe, Asia	- total dairy<br>- milk<br>- cheese<br>- yogurt	Total dairy products: H vs L exposure category<br><br>Milk: H vs L exposure category<br><br>Cheese: H vs L<br><br>Dairy products intake among women with H vs L BMI	Endometrial cancer risk, with a focus on the impact of BMI.	neutral: total dairy, milk, cheese	Up to February 26, 2017.	health outcome only present in women
Dietary Protein Consumption and the Risk of Type 2 Diabetes: A Systematic Review and Meta-Analysis of Cohort Studies (Tian, 2017)	99	SR/MA	Type 2 Diabetes Mellitus	The aim of the review was to investigate the association between dietary protein consumption and the risk of type 2 diabetes mellitus (T2DM) through a systematic review and meta-analysis of cohort studies. The review also aimed to compare the association between different food sources of protein and the risk of T2DM.	Prospective Cohort Studies	11 total dairy,<br>7 milk,<br>7 yogurt	483,174	USA, Europe, Asia, Australia, Finland	total dairy, whole milk, yogurt	high vs low consumption	Risk of Type 2 Diabetes Mellitus (T2DM) associated with specific high-protein food sources such as total dairy products, whole milk, yogurt	decreased association: total dairy, whole milk, yogurt	up to July 2017	Yes
Dietary Protein Sources and Incidence of Breast Cancer: A Dose-Response Meta-Analysis of Prospective Studies (Wu, 2017)	31	SR/MA	Breast Cancer	The aim of the review was to investigate the association between different dietary protein sources and breast cancer risk.	cohort studies case-control randomized controlled trials	46	7,606,009	United States, Europe, Asia, North America, Western Europe	Total Milk<br><br>Yogurt	high/low intake and dose/response	Risk of breast cancer	protective: yogurt<br>neutral: total milk	up to Dec 2015	Only incl. women
Dose-Dependent Effect of Intake of Fermented Dairy Foods on the Risk of Diabetes: Results From a Meta-analysis (Zhang, 2022)	33	SR/MA	type 2 diabetes	To estimate the overall risk of diabetes mellitus associated with the intake of fermented dairy foods and investigate any dose-dependent effects of higher intake of fermented dairy foods on diabetes risk.	cohort studies case-control studies	15	485,992	USA, Japan, UK, Spain, The Nether-lands, Sweden, Korea, Denmark	dairy	High/low intake	Risk of diabetes	protective: cheese, fermented dairy	January 1980 to Dec 2020	No

Effect of Milk and Cultured Milk Products on Type 2 Diabetes: A Global Systematic Review and Meta-analysis of Prospective Cohort Studies (Mohan, 2023)	34	SR/MA	type 2 diabetes	The aim of the review was to investigate the associations between various dairy products and incident type 2 diabetes (T2D) globally, with a focus on total dairy products, fermented dairy, plain yogurt, low and high fat dairy products, milk, and cheese, stratified by ethnicity and age groups.	prospective cohort studies	27	1,771,026	United States of America, Europe, Australia, Asia	- dairy<br>- milk<br>- cheese<br>- yogurt	Total dairy vs. no/lower dairy consump-tion	incident T2D	protective: Total dairy, fermented dairy, yogurt<br><br>neutral: milk, cheese and other dairy products	January 2000 - March 2022	No
Effects of Dairy Intake on Markers of Cardiometabolic Health in Adults: A Systematic Review with Network Meta-Analysis (Kiesswetter, 2023)	35	SR/MA	cardiometa bolic health	The aim of the review was to compare the effects of different dairy products on markers of cardiometabolic health in the general healthy adult population. The associations investigated were the effects of dairy intake and specific dairy products on markers of cardiometabolic health.	RCTs	19	1,427	North America, Scandin-avia, Asia, and Turkey.	- dairy<br>- milk<br>- yogurt<br>- kefir<br>- cheese<br>- mixed dairy products	- high dairy intake versus low dairy/contr ol intake<br>- specific dairy products (milk, yogurt, kefir, and mixed dairy products)	Anthropomet ric outcomes), blood lipids, glycemic control, and systolic blood pressure	neutral: all dairy on blood pressure, all dairy on body weight	inception to 23 Sept 2022.	No
Effects of dairy products, calcium and vitamin D on ovarian cancer risk: a meta-analysis of twenty-nine epidemiological studies (Liao, 2020)	36	SR/MA	Cancer, ovarian	To assess the associations between dairy products, calcium, and vitamin D intake with ovarian cancer risk through an updated meta-analysis.	- 18 Case-control studies<br>- 11 Cohort/nest ed case-control studies	29	963,604	Worldwide	- dairy (including total dairy products)<br>- milk (including whole milk and low-fat milk)<br>- cheese	- Highest vs. lowest intake - 100 g/d increment in total dairy products<br>- 100 g/d increment s	Ovarian cancer risk.	increased risk for total dairy,<br><br>decreased for cheese	inception to Dec 2019.	health out-come only in women

Effects of Milk and Dairy Products on the Prevention of Osteoporosis and Osteoporotic Fractures in Europeans and Non-Hispanic Whites from North America: A Systematic Review and Updated Meta-Analysis (Matía-Martín, 2019)	37	SR/MA	Osteoporosis	The aim of the review is to update the evidence regarding dairy intake, osteoporotic fracture (OF) risk, and prospective bone mass density (BMD) evolution in Europeans and non-Hispanic whites from North America.	Prospective Cohort studies and case-control studies	9	377,727	North America - only Caucasian	- dairy<br>- milk<br>- cheese<br>- cream<br>- yogurt	- H vs L dairy intake<br>- Different types of dairy products<br>- Dose-response relationship for each increment in dairy intake	Osteoporotic fracture (OF) at any site, Hip fractures, Vertebral fractures, Bone mineral density (BMD) changes at different sites (hip, trochanter, radius)	all dairy products: conflicting cheese and yogurt: protective milk: neutral	1 Jan, 2000 to 30 April, 2018	No
Fermented Dairy Food Intake and Risk of Colorectal Cancer: A Systematic Review and Meta-Analysis (Liang, 2022)	46	SR/MA	Cancer - colorectal	The aim of the review was to investigate the associations between fermented dairy food intake, specifically yogurt and cheese, and the risk of colorectal cancer.	- Case-Control studies<br>- Prospective Cohort Studies	17	1,325,780	Morocco, United States, Canada, Japan, and 10 different European countries	yogurt, cheese	Comparison: High vs. low consump-tion of cheese and yogurt	CRC risk and mortality	Protective CRC: cheese<br><br>Protective rectal cancer: cheese, yogurt<br><br>neutral: CRC: yogurt neutral: mortality: yogurt	inception until July 2021	Yes
Fermented dairy foods intake and risk of cancer (Zhang, 2019)	38	SR/MA	cancer: bladder, colorectal, esophageal, prostate, renal, and ovarian.	The aim of the review was to investigate the association between fermented dairy foods intake and cancer risk, including the overall cancer risk and specific associations with different cancer types.	Cohort studies and case-control studies	61	1,962,774	Worldwide	dairy<br>yogurt<br>cheese<br>probiotics	Intake of fermented dairy foods, yogurt, & cheese versus cancer risk and specific types of cancer.	cancer risk, with a focus on specific types of cancer such as bladder cancer, colorectal cancer, esophageal cancer, prostate cancer, renal cancer, and ovarian cancer	all fermented: protective: all cancers, colorectal, bladder, esophageal<br><br>cheese protective: CRC<br><br>yogurt protective: CRC, bladder	January 1980 to July 2018	No

Fermented dairy foods intake and risk of cardiovascular diseases: A meta-analysis of cohort studies (Zhang, 2019)	47	SR/MA	Cardiovascular Diseases	The aim of the review was to investigate the association between fermented dairy foods intake and the risk of cardiovascular diseases (CVD) through a meta-analysis of published cohort studies.	Prospective Cohort Studies	10	Participant count: 385,122	Sweden, UK, Netherlands, Australia, USA	yogurt or cheese	Not explicitly stated. Consump-tion gathered by food questionnaire; may be H vs L.	Risk of cardiovascular diseases (CVD)	protective: dairy, cheese, yogurt: CVD	January 1980 to September 2018	No
Fermented Milk Products and Bone Health in Postmenopausal Women: A Systematic Review of Randomized Controlled Trials, Prospective Cohorts, and Case-Control Studies (Ong, 2020)	48	SR/MA	Bone health	The aim of the review was to investigate the association between fermented milk products (FMPs) and bone health indicators in postmenopausal women, focusing on outcomes such as fracture incidence, bone mineral density (BMD), BMD T-score, and percentage change in bone turnover markers.	RCTs/Randomized Controlled Trials<br><br>Prospective Cohort Studies<br><br>Case-Control studies	9 studies total in qualitative synthesis<br><br>3 in meta-analysis	103,288	Multi-national	yogurt, cheese	consumption of FMPs to that of non FMPs, low consumption, no consumption, or placebo, were included.	incidence of vertebral or nonvertebral fractures, percentage change from baseline in BMD of the lumbar spine, the total hip, or the femoral neck, BMD T-score of the lumbar spine, total hip, or femoral neck, and percentage change in bone turnover markers.	protective: hip fracture: yogurt<br><br>neutral: hip fracture: cheese<br><br>conflicting: osteoporosis: cheese	January 1947 – January 2019	Only women included in study
Food groups and risk of all-cause mortality: a systematic review and meta-analysis of prospective studies (Schwingshackl, 2017)	49	SR/MA	Mortality	The aim of the review was to investigate the associations between the intake of 12 major food groups and the risk of all-cause mortality through a systematic review and meta-analysis of prospective studies.	Prospective Cohort Studies, Case-Cohort Studies, Follow-ups of RCTs, Case-Control studies	27 studies on dairy products	1,036,899	Europe, North America, Asia, Australia	dairy products	high-intake versus low-intake categories groups, & linear and nonlinear dose-response relation-ships.	Risk of all-cause mortality associated with the intake of specific food groups	neutral	Up to December 2016	Yes, in supplemental documents

Food groups and risk of colorectal cancer (Schwingshackl, 2018)	50	SR/MA	Cancer - colorectal	The aim of the review was to investigate the associations between the intake of 12 major food groups and the risk of colorectal cancer.	prospective studies including cohort studies, case-cohort studies, follow-up of RCTs, and nested case-control studies.	For dairy as exposure: (11 colon, 7 rectal)	16,910 cases with dairy as exposure	North America, Asia, Australia	dairy	High vs. low intake of dairy products	Risk of colorectal cancer (CRC)	protective: dairy	May 2015 to April 2017	Yes, in supplemental documents
Food groups and risk of coronary heart disease, stroke and heart failure: A systematic review and dose-response meta-analysis of prospective studies (Bechthold, 2019)	51	SR/MA	CHD, Stroke, heart failure	The aim of the review was to investigate the associations between the intake of 12 major food groups and the risk of CHD, stroke, and HF through a systematic review and meta-analysis of prospective studies.	Prospective Cohort Studies<br><br>Case-cohort Studies<br><br>Nested Case-Control Studies<br><br>Follow-up Studies of RCTs	total 123; 24 about dairy	13 studies, 15,790 cases on dairy and CHD<br>12 studies and 16,887 cases on dairy and stroke	Europe, Asia, Australia	Dairy (among others)	high/low intake, linear and non-linear dose/response	risk of CHD, stroke, and heart failure (HF) in relation to the intake of 12 major food groups,	neutral: dairy on stroke, dairy on CHD	from 1995 to March 2017.	Yes, in supplemental documents
Food Groups and Risk of Hypertension: A Systematic Review and Dose-Response Meta-Analysis of Prospective Studies (Schwingshackl, 2017)	52	SR/MA	Hypertension	The aim of this systematic review and meta-analysis was to summarize the evidence on the relation of the intakes of 12 major food groups with the risk of hypertension.	Prospective Cohort Studies, Case-Cohort Studies, Nested Case-Control Designs Follow-ups of RCTs	9 for dairy products	Total participants included in review: 1,052,670	Asia, Australia, UK, Europe, North America	dairy	highest and lowest intake categories, and linear dose-response	Risk of hypertension associated with the intake of different food groups, assessed through linear & nonlinear dose-response meta-analyses.	protective: dairy	Studies published until June 2017.	No

Food Groups and Risk of Overweight, Obesity, and Weight Gain: A Systematic Review and Dose-Response Meta-Analysis of Prospective Studies (Schlesinger, 2019)	53	SR/MA	Body Composition	The aim of the review was to investigate the associations between the intake of 12 predefined food groups and the risk of general overweight/obesity, abdominal obesity, and weight gain through a systematic review and meta-analysis of prospective observational studies.	Prospective Cohort Studies, case-cohort studies, follow-up of RCTs and nested case-control studies	25 total studies 43 articles	Not available	Multi-national	dairy	high compared with low intake and dose-response	General overweight/ obesity, abdominal obesity, or weight gain as a dichotomous endpoint;	neutral: overweight/ obesity, abdominal obesity, weight gain	Until August 2018	no
Food groups and risk of type 2 diabetes mellitus: a systematic review and meta-analysis of prospective studies (Schwingshackl, 2017)	54	SR/MA	Type 2 Diabetes Mellitus	The aim of the review was to investigate the associations between the intake of 12 major food groups and the risk of type 2 diabetes (T2D).	prospective design studies (cohort studies, nested case–control studies, case-cohort studies, follow-up of RCTs)	Dairy products: 21 studies	44,474 T2DM cases included regarding dairy as exposure	Asia, Australia, USA, Europe	dairy	high versus low intake, dose-response associations	the risk of type 2 diabetes (T2D)	protective: dairy: T2D	Up to February 2017	Yes, in supplemental documents
Food of animal origin and risk of non-Hodgkin lymphoma and multiple myeloma: A review of the literature and meta-analysis (Caini, 2016)	55	SR/MA	Cancer - non-Hodgkin lymphoma & Multiple Myeloma	The associations investigated in the systematic review were the consumption of red meat, processed meat, white meat, fish and seafood, dairy products, milk, cheese, and eggs in relation to the risk of non-Hodgkin lymphoma and its major subtypes (diffuse large B-cell lymphoma, follicular lymphoma, chronic lymphocytic leukaemia/ small lymphocytic lymphoma) and multiple myeloma among adults.	human observational studies with a cohort, case-control or case-cohort study design	33, unclear how many on dairy	Not available	Europe, Northern and Southern America, and Asia; multinational	Dairy products (all), cheese, and milk	high/low intake	Non-Hodgkin lymphoma and its major subtypes (diffuse large B-cell lymphoma, follicular lymphoma, chronic lymphocytic leukaemia/ small lymphocytic lymphoma) and multiple myeloma	harmful: dairy: Non-Hodgkin lymphoma	Up to Nov 30, 2014	No

High vs. low-fat dairy and milk differently affects the risk of all-cause, CVD, and cancer death: A systematic review and dose-response meta-analysis of prospective cohort studies (Naghshi, 2021)	94	SR/MA	Mortality – all-cause, CVD, cancer	We conducted a comprehensive dose-response meta-analysis of prospective cohort studies to examine the association between total, low-fat, and high-fat dairy consumption and risk of all-cause, CVD, and cancer mortality	prospective observation al studies	47 papers:	2,967,447	Worldwide	High vs. Low-fat dairy and milk	High vs. low and dose-response	Mortality – all-cause, CVD, cancer	Total dairy: Neutral: all-cause mortality,<br><br>Conflicting results: CV mortality<br><br>Total milk: Neutral: all-cause mortality, CV mortality	up to February 2020	yes
Higher Yogurt Consumption Is Associated With Lower Risk of Colorectal Cancer: A Systematic Review and Meta-Analysis of Observational Studies (Sun, 2021)	56	SR/MA	Cancer - colorectal	The aim of the review is to investigate the association of yogurt consumption with the risk of colorectal cancer (CRC) in the general population. The review aims to clarify the inconsistent findings in the literature and provide an updated systematic review and meta-analysis on this topic. The research question specifically focuses on whether higher yogurt intake is related to a lower risk of CRC.	case-control, cohort	16	1,129,035	Europe, North America, Asia, Africa, multiple European countries	yogurt	highest vs. the lowest categories of yogurt intake	Risk of colorectal cancer (CRC)	protective: yogurt: CRC	Until July 2021	Yes
Influence of dairy products consumption on oral cancer risk: A meta-analysis (Rodriguez-Archilla, 2023)	57	SR/MA	Cancer - oral	This study aimed to determine the influence of dairy product consumption (milk, cheese, yogurt, butter) on oral cancer risk.	case-control studies	21 studies total:<br>21 about milk<br>14 about cheese<br>8 about yogurt	59,271 participants	Worldwide	regular milk, cheese, and yogurt consumpti on	Not clear – reg. consump-tion is mentioned Dose-response/ quantity consider-ed in some studies.	oral cancer	protective: milk, cheese, yogurt	Up to October 2022	No

Intake of dairy products and associations with major atherosclerotic cardiovascular diseases: a systematic review and meta-analysis of cohort studies (Jakobsen, 2021)	58	SR/MA	Cardiovascular diseases	The aim of the review is to investigate the associations between total dairy product intake and intake of dairy product subgroups with the risk of major atherosclerotic cardiovascular diseases in the general adult population, focusing on specific types of dairy products such as high-fat milk, cheese, yogurt, and butter in relation to coronary heart disease (CHD) and ischemic stroke.	Prospective Cohort studies	18 studies were included<br>- 13 for CHD<br>- 7 for ischemic stroke	2,263,815	Europe, North America, Asia, multinational	- dairy<br>- milk<br>- cheese<br>- yogurt	- High versus low intake<br>- Dose-response (higher intake in grams per day)	Risk of major atherosclerotic cardiovascular diseases, specifically coronary heart disease (CHD) and stroke	Neutral: yogurt: CHD & stroke; milk: CHD, cheese: stroke, total dairy: CHD & Stroke<br><br>Conflicting: milk: stroke<br><br>Protective: cheese: CHD	up to August 15, 2019	No
Intake of milk and other dairy products and the risk of bladder cancer: a pooled analysis of 13 cohort studies (Acham, 2020)	59	Pooled MA	Cancer - bladder	The aim of the review is to investigate the associations between milk and other dairy products consumption and the risk of developing bladder cancer by pooling data from cohort studies.	Cohort studies	13	597,227	USA, Sweden, the Netherlands, Australia, Denmark, France, Germany, Greece	- dairy<br>- milk<br>- cheese<br>- yogurt	Ever vs. never intake<br>Consumption per cup per day<br>- Tertiles of dairy product consumption vs no consumption<br>Dose/response for yogurt consumption	Risk of developing bladder cancer	neutral: milk, total dairy, cheese<br>protective: yogurt	n/a	Yes, but not presented

Intake of Various Food Groups and Risk of Breast Cancer: A Systematic Review and Dose-Response Meta-Analysis of Prospective Studies (Kazemi, 2021)	60	SR/MA	Cancer - breast	The aim of the review was to summarize the associations between various food groups and the risk of breast cancer, assessing both linear and nonlinear dose-response relationships.	Prospective Cohort studies<br>- Case-cohort studies<br>- Nested case-control studies<br>- Follow-up studies of randomized controlled trials (RCTs)	Dairy: 10<br>Milk: 13<br>Yogurt: 6<br>Cheese: 10	131,898	Asia, Europe, North America, Australia	- dairy<br>- milk<br>- yogurt<br>- cheese	- Linear dose-response for incre-ments of intake<br><br>Nonlinear dose-response for varying levels of intake	Risk of breast cancer	protective: cheese conflicting: milk	up to March 2020.	Only women included in study
Is consuming yogurt associated with weight management outcomes? Results from a systematic review (Eales, 2016)	61	SR	Body Composition	The aim of the review is to investigate the effects of yogurt consumption on weight-related outcomes in apparently healthy adults, focusing on associations with lower body mass index, lower body weight/weight gain, smaller waist circumference, and lower body fat in epidemiological studies. The review also aims to identify data on the effectiveness of yogurt consumption in specific subgroups such as people at risk of diabetes and people from different ethnic groups.	Cohort studies: 6<br>RCTs: 6<br>Controlled trial (CT): 1<br>Cross-over studies: 2<br>Prospective cohort study: 1<br>Retro-spective population-based cohort studies: 5<br>Cross-sectional studies: 7	28	Not available	USA, Korea, Malaysia, Spain, Australia, Canada, France	yogurt	yogurt consumption with low or no yogurt consumption, placebo, non-yogurt substances, and non-yogurt interventions.	Change in body weight, Change in waist circumference, Change in body fat, BMI, lean body mass, risk/proportion of overweight/obesity.	protective: BMI	Up to October 2014	No

Long-Term Consumption of 10 Food Groups and Cardiovascular Mortality: A Systematic Review and Dose Response Meta-Analysis of Prospective Cohort Studies (Bhandari, 2023)	63	SR/MA	Mortality - cardiovascular	The aim of the review is to evaluate the relationship between the long-term consumption of 10 food groups and cardiovascular mortality.	Prospective Cohort studies	22 total, 4 on dairy	29,990 cardiovascular mortality cases included in studies on dairy product intake	Australia, Sweden, USA	dairy	- High versus low intake<br>- Dose-response (incremental increase in consumption)	cardiovascular mortality	neutral: cardiovascular mortality	January 2000 - January 2022	No, but discussed
Meat, fish, dairy products and risk of hematological malignancies in adults. a systematic review and meta-analysis of prospective studies (Sergentanis, 2019)	65	SR/MA	Cancer - leukemia, acute myeloid leukemia, non-Hodgkin lymphoma (NHL) and small lymphocytic lymphoma/ chronic lymphocytic leukemia (CLL/SLL).	The aim of the review was to examine the association of meat, fish, and dairy product consumption with the risk of hematological neoplasms in adults.	Cohort studies	13 total, 3 studies on milk and dairy	1,480,986 participants	USA, Europe, Japan	Milk and dairy product consumption	Not available	risk of hematological malignancies	neutral: hematological malignancies	Until August 31, 2016	no
Meta-Analysis of Milk Consumption and the Risk of Cognitive Disorders (Wu, 2016)	66	SR/MA	Dementia, Alzheimer's cognitive decline/ impairment	The aim of the review was to investigate the association between milk consumption and cognitive disorders, specifically Alzheimer's disease, dementia, and cognitive decline/impairment, by conducting a systematic review and meta-analysis of observational studies.	cohort studies, cross-sectional studies	7	10,941	Australia, Japan, Africa, USA, China, France, Japan	milk intake or milk combined with other dairy products	H vs L level of milk intake, as well as between different population: Asian, African, Caucasian	Risk of cognitive disorders, including Alzheimer's disease, dementia, and cognitive decline/impairment	protective	Inception to October 2016	Yes

Milk and dairy consumption and risk of cardiovascular diseases and all-cause mortality: dose-response meta-analysis of prospective cohort studies (Guo, 2017)	67	SR/MA	Cardiovascular diseases & all-cause mortality	The aim of the review was to conduct a dose-response meta-analysis of milk and dairy products with all-cause mortality, coronary heart disease (CHD), or cardiovascular disease (CVD) based on prospective cohort studies.	Prospective Cohort studies	29	938,465	multinational: United States, Europe, Asia (Japan and Taiwan), Australia	- dairy<br>- milk<br>- cheese<br>- yogurt<br>- fermented dairy<br>High-fat dairy, low-fat dairy	Total daily intake	all-cause mortality, CHD, CVD	neutral: total dairy & milk: mortality, CHD, CVD<br><br>protective: total fermented dairy: mortality & CVD risk<br>protective: cheese: CVD risk<br><br>neutral: yogurt: all-cause mortality, CHD, CVD	Up to Sept 2016.	Yes
Milk and Dairy Product Consumption and Bladder Cancer Risk: A Systematic Review and Meta-Analysis of Observational Studies (Bermejo, 2019)	68	SR/MA	Cancer - bladder	The aim of the review was to investigate the association between milk and dairy product consumption and bladder cancer risk by conducting a systematic review and meta-analysis of observational studies.	- Case-Control studies<br>- Prospective Cohort Studies	26	595,698	Worldwide	dairy<br>milk<br>cheese<br>kefir<br>buttermilk<br>koumiss<br>yogurt<br>probiotic yogurt	Medium vs. low consumption and high vs. low consumption for each type of dairy product.	Risk of bladder cancer	protective: total dairy<br>protective: milk<br>protective: fermented dairy<br>neutral: cheese	Inception to April 2018	No
Milk and dairy products consumption and the risk of oral or oropharyngeal cancer: a meta-analysis (Yuan, 2019)	69	SR/MA	Cancer - oral	The aim of the review was to investigate the association between milk and dairy products consumption and the risk of oral or oropharyngeal cancer through a meta-analysis.	Observational study, case-control design	12	50,777	Europe, Asia, America	milk and dairy products consumption	amount of milk/dairy consumed	risk of oral or oropharyngeal cancer in relation to milk and dairy products consumption	protective: milk and dairy: oral or oropharyngeal cancer risk (pooled results)<br><br>Some heterogeneity in different populations	Inception until June 30, 2019	Yes

Milk and yogurt intake and breast cancer risk - A meta-analysis (Chen, 2019)	70	SR/MA	Cancer - breast	The aim of the review was to investigate the association between dairy food intake, including milk and yogurt, and the risk of breast cancer through a meta-analysis of case-control studies.	Case-Control studies	8	Not available	United States, France, the Netherlands, Norway, Finland, Japan, Europe	dairy products - low fat/skim milk and yogurt	Intake of low-fat/skim milk, whole milk, and yogurt consumption versus seldom/never consumed in relation to the risk of breast cancer	Risk of breast cancer	neutral: milk, yogurt	up to June 2, 2009.	Only women included in study
Milk Consumption and Mortality from All Causes, Cardiovascular Disease, and Cancer: A Systematic Review and Meta-Analysis (Larsson, 2015)	71	SR/MA	Cardiovascular disease, all-cause mortality, cancer	The aim of the review was to investigate the association between non-fermented and fermented milk consumption with mortality from all causes, cardiovascular disease, and cancer.	Prospective Cohort studies	12 articles based on 13 prospective studies	367,505	UK, Scotland, Sweden, US, Netherlands, Japan, Australia, multinational	-milk<br>-yogurt<br>-soured milk (fermented)	dose-response	Mortality from all causes, cardiovascular disease, and cancer	Inconsistent /conflicting: milk, yogurt, fermented dairy and mortality	Until August 2015	No
Milk Intake in Early Life and Later Cancer Risk: A Meta-Analysis (Gil, 2022)	72	SR/MA	Cancer - breast, prostate, colorectal	The aim of the review was to investigate the relationship between milk intake in early life and later cancer risk through a meta-analysis of observational studies.	- Case-Control Studies<br>- Cohort Studies	Breast: 9<br>Prostate: 3<br>Colorectal: 2<br>breast, prostate & CRC: 1	Not available	UK, New Zealand, Norway, USA, Sweden, Iceland, China	milk and dairy products during childhood and adolescence	adolescent dairy intake, fat content of dairy intake (low-fat, regular), and life stage of dairy intake.	Breast cancer risk, prostate cancer risk, and colorectal cancer risk	neutral: milk: breast, prostate, colorectal cancers	to Dec 2021	No
Milk, Yogurt, and Lactose Intake and Ovarian Cancer Risk: A Meta-Analysis (Liu, 2015)	73	SR/MA	Cancer - ovarian	The aim of the review was to assess the association between intakes of milk, lactose, and milk products (yogurt) and the risk of ovarian cancer through a meta-analysis.	Case-control studies	19	Not available	United States, Canada, Australia, Sweden, Mexico, North America, Western Europe, Denmark	- milk (including low-fat/skim milk and whole milk)<br>- yogurt	-highest versus lowest category (dose/response)	risk of ovarian cancer	neutral: milk neutral: yogurt	up to February 2014.	No, outcome only in women

Milk/dairy products consumption and gastric cancer: an update meta-analysis of epidemiological studies (Wang, 2018)	74	SR/MA	Cancer - gastric	The aim of the review was to update and quantitatively reassess the association between dairy products consumption and gastric cancer by conducting a meta-analysis of published cohort and case-control studies.	Case-control study, Cohort study	34	87,275	United States, Europe, Asia, Latin America, Turkey	milk/dairy	Highest versus lowest categories of dairy consump-tion	gastric cancer risk	harmful: dairy	Up to March 2016	No
Role of Dairy Foods, Fish, White Meat, and Eggs in the Prevention of Colorectal Cancer: A Systematic Review of Observational Studies in 2018-2022 (Alegria-Lertxundi, 2022)	76	SR	Cancer - colorectal	The review aimed to investigate the associations between the consumption of dairy products, fish, white meat, and eggs and the risk of colorectal cancer (CRC) based on observational studies in adults..	- Case-control studies<br>- Prospective Cohort Studies	Number of Studies included: 21 (14 cohort, 7 case-control)	3,309,275	Europe, China, United States, Iran, Spain	various types of dairy categories (total milk, cheese, total dairy, yogurt, fermented dairy,	high/low intake or certain quantity versus never	risk of colorectal cancer (CRC) in observationa l studies.	protective: total dairy<br><br>neutral: milk and cheese<br><br>conflicting: yogurt	January 2018 to July 2022	No
Role of dietary patterns and factors in determining the risk of knee osteoarthritis: A meta-analysis (Xu, 2022)	77	SR/MA	Osteoarthritis	The aim of the review was to investigate the associations between different dietary patterns or factors and the risk of knee osteoarthritis.	longitudinal prospective cohorts or their sub-studies, cross-sectional	There were 15 studies included in the review, 2 included studies had dairy as exposure	Participant count: 97,157	Not available	dairy	high use of dairy products versus low use	Risk of knee osteoarthritis	protective: dairy	inception date to Dec 2020	No
Role of milk and dairy intake in cognitive function in older adults: a systematic review and meta-analysis (Lee, 2018)	78	SR/MA	dementia, cognitive decline/imp airment, cognitive function	The aim of the review is to examine the effects of varying levels of milk intake alone or in combination with other dairy products on the outcomes of cognitive function and disorders in adults.	RCT/Rando mized Controlled Trials<br><br>Prospective Cohort studies	1 RCT<br>7 Cohort studies	25,317	France, U.S.	dairy milk and dairy consumpti on, varied	- H vs L dairy diet<br>- doses of milk intake alone or w/ other dairy products<br>- Regular consumptio n vs. rare consump-tion of whole milk<br>- H vs L milk intake	cognitive function, cognitive decline, AD	neutral	Inception through October 2017	No

Subgroup dairy products consumption on the risk of stroke and CHD: A systematic review and meta-analysis (Gholami, 2017)	79	SR/MA	stroke & CHD	The aim of the review was to investigate the associations between dairy product consumption and cardiovascular diseases, specifically stroke and coronary heart disease, by integrating the results of several prospective cohort studies. The review aimed to determine the effects of different dairy products on CVD.	Prospective cohort studies	11	Total: 653,164 participants	The Nether-lands, Sweden, UK, Finland, Japan	Dairy products consumption.	different types of dairy products (milk, cheese, cream, and butter)	Risk of cardiovascular diseases (CVD), specifically stroke and coronary heart disease (CHD).	neutral: milk: stroke<br><br>protective: cheese: stroke<br><br>neutral: milk, cheese: CHD	Up to Sept 2014	Yes
Systematic review and meta-analysis: dairy consumption and hepatocellular carcinoma risk (Yang, 2017)	80	SR/MA	Cancer - liver	The aim of the review is to assess the association between dairy consumption and hepatocellular carcinoma (HCC) risk, including the consumption of overall dairy and specific dairy products.	cohort, case-control	8	1,084,666	Japan, Europe, USA, China, Italy, Greece, Serbia	dairy consump-tion including all dairy, milk, yogurt, and cheese	Consump-tion of overall dairy vs no dairy consump-tion, consump-tion of specific dairy products vs no consumption, H vs L levels of dairy consump-tion	hepatocellular carcinoma (HCC) (liver cancer)	harmful: dairy and cheese: HCC risk<br><br>neutral: milk and yogurt: HCC risk	Up to Sept 20, 2015	No
The association between breast cancer and consumption of dairy products: a systematic review (Arafat, 2023)	82	SR	Cancer - breast	The aim of the review was to assess the association between dairy food consumption and the development of breast cancer, to investigate the link between consuming milk or other dairy foods and the risk of breast cancer, and to summarize the findings on the association between different types of dairy products and breast cancer risk.	The study designs of the included studies are:<br><br>Prospective Cohort Studies<br><br>Retrospective Studies<br><br>Cross-Sectional Studies	18: 9 Prospective, 7 Retrospective and 2 Cross-Sectional studies	680,556	United States, Iran, Spain, the Netherlands, France, China, Korea, Japan, Poland, Algeria, Europe	cheese, milk, yogurt	servings/ amount per day	Risk of developing breast cancer	protective: dairy<br><br>protective: yogurt	Jan 1 2008 to Jan 31 2022	No, primarily women suffer from breast cancer

The Association between Dairy Intake and Breast Cancer in Western and Asian Populations: A Systematic Review and Meta-Analysis (Zang, 2015)	83	SR/MA	Cancer - breast	The aim of the review was to examine the association between dairy consumption and breast cancer risk, including the dose-response relationship, different types of dairy products, and the impact on both Western and Asian populations.	prospective cohort and case-control studies	22 prospective cohort studies and 5 case-control studies were included in the review.	1,600,312	United States, Europe, Japan, China, Iran	dairy, yogurt, milk, cheese	Different levels of dairy consump-tion (high, modest, low) compared through a dose-response analysis.	risk of breast cancer compared to low dairy consumption	protective: all dairy, yogurt<br><br>neutral: milk, cheese	Jan 1989 to Jan 2014	No, primarily women suffer from breast cancer
The association between dairy products consumption and prostate cancer risk: a systematic review and meta-analysis (Zhao, 2023)	84	SR/MA	Cancer - prostate	The aim of the review is to investigate the relationship between the consumption of dairy products and the risk of prostate cancer through a systematic review and meta-analysis.	prospective cohort studies.	33	Participant count: 4,212,923	Multi-national: USA, UK, multiple countries of Europe, Japan	dairy consump-tion including total dairy, milk, butter, cheese, ice cream, yogurt, and other dairy products	different levels of consump-tion of total dairy products, total milk, cheese, butter, and whole milk	prostate cancer incidence	harmful: total dairy, total milk, cheese<br>protective: whole milk<br>neutral: yogurt, skim milk	Up to March 2021	Only males suffer from prostate cancer
The Effect of Daily Fluid Management and Beverages Consumption on the Risk of Bladder Cancer: A Meta-analysis of Observational Study (Hong, 2018)	85	SR/MA	Cancer - bladder	The aim of the review was to explore the potential relationship between daily fluid intake and the risk of bladder cancer by conducting a meta-analysis.	Prospective Cohort studies, Case-control studies	54<br>12 including milk as exposure	2,208,863	North America, Europe, Asia	milk	H vs L intake of specific beverages	risk of bladder cancer.	protective: milk	Inception to June 2018	Yes
The effect of dairy consumption on the prevention of cardiovascular diseases: A meta-analysis of prospective studies (Gholami, 2017)	86	SR/MA	Cardiovas-cular Diseases	The aim of the review was to evaluate the effect of dairy consumption on cardiovascular diseases, including stroke and coronary heart disease. The associations investigated in the systematic review were the relationship between total dairy intake and CVD, stroke, and CHD.	Prospective Cohort Studies	27	Total: 982,067	Worldwide	dairy products	total dairy intake	Cardiovascular diseases (CVD) incidence and mortality, Coronary heart disease (CHD) incidence and mortality, Stroke incidence and mortality	protective: total dairy intake and CVD, stroke<br><br>neutral: dairy intake and CHD	Up to Sept 2014	Yes

The Effects of Dairy Intake on Insulin Resistance: A Systematic Review and Meta-Analysis of Randomized Clinical Trials (Sochol, 2019)	87	SR/MA	body weight	The aim of the review was to investigate the associations between dairy intake, especially low-fat dairy products, and homeostatic model assessment of insulin resistance (HOMA-IR), waist circumference, and body weight in randomized clinical trials.	Systematic review and meta-analysis of randomized clinical trials with risk of bias assess-ment	30	2,900	United States	dairy	Different levels of dairy intake, such as high dairy intake versus low dairy intake	HOMA-IR, waist circum-ference, body weight	protective: body weight	Inception to July 2016	No
The relationship between dairy products intake and breast cancer incidence: a meta-analysis of observational studies (He, 2021)	88	SR/MA	Cancer - breast	The aim of the review is to investigate the relationship between dairy intake and breast cancer incidence through a meta-analysis.	- Cohort studies<br>- Case-control studies	36	1,019,232	Europe, North America, Asia, South America	dairy<br>milk<br>cheese<br>fermented dairy products<br>non-fermented dairy products<br>yogurt (as part of fermented dairy products)	-Total dairy products vs. no or rare consumption<br>Fermented dairy products vs. non-fermented dairy products	different types of breast cancer	protective: total dairy<br>protective: ER+ and PR+ breast cancer dairy<br>protective: fermented dairy in post-menopausal population<br>neutral: non-fermented dairy	Up to January 2021	No, primarily women suffer from breast cancer
Yogurt Intake and Gastric Cancer: A Pooled Analysis of 16 Studies of the StoP Consortium (Collatuzzo, 2023)	89	Pooled MA	Cancer – gastric	The aim of the review was to investigate the association between yogurt intake and gastric cancer risk, including anatomical and histological subtypes, by pooling data from 16 international studies. The review aimed to explore the potential role of yogurt in modifying gastrointestinal disease risk. The review also highlighted the under-investigated association between yogurt and gastric cancer.	case-control studies	16	20,459	Italy, Portugal, Spain, Greece, Finland, Japan, Russia, Mexico, USA, Brazil	yogurt consumption	dose/response, consumed /not consumed, high/low intake	gastric cancer (GC) risk	neutral: overall (yogurt)	n/a	Yes

Yogurt consumption and risk of mortality from all causes, CVD and cancer: a comprehensive systematic review and dose-response meta-analysis of cohort studies (Tutunchi, 2023)	90	SR/MA	Mortality - all causes, CVD, and cancer	The aim of the review is to quantify the dose-response relation between yogurt consumption and the risk of mortality from all causes, CVD, and cancer.	cohort studies	18	896,871	USA, UK, the Nether-lands, Australia, Japan, Italy, Iran, multinational	yogurt	highest vs. lowest intakes of yogurt	Deaths from all causes, Cardiovas-cular disease (CVD) mortality	protective: all-cause mortality<br><br>protective: CVD mortality<br><br>neutral: cancer mortality	Inception to August 2022	Yes
Yogurt Intake Reduces All-Cause and Cardiovascular Disease Mortality: A Meta-Analysis of Eight Prospective Cohort Studies (Gao, 2020)	91	SR/MA	Mortality - all causes, CVD, cancer	The aim of the review was to investigate the association between yogurt intake and all-cause mortality as well as cardiovascular disease and cancer mortality.	Prospective Cohort Studies	8 studies included (7 all-cause mortality 4 CVD mortality 3cancer mortality)	235,676	Worldwide	yogurt	High vs. lower levels of yogurt intake	All-cause mortality, CVD mortality, cancer mortality	protective: all-cause mortality<br><br>protective: CVD mortality<br><br>neutral: cancer mortality	Until October 2018	No

Supplemental Table B: Extraction table of World Cancer Research Fund included papers

Title	PAPER ID	Pub year	Outcome	Intervention/ Exposure	Outcome measured	Strong evidence	Convincing evidence	Probable evidence	Limited evidence	Limited/ suggestive evidence	Limited - no conclusion	Substantial effect on risk unlikely
Diet, nutrition, physical activity and endometrial cancer	102	2018	Endometrial cancer	dairy, milk	endometrial cancer incidence						milk and dairy products	
Diet, nutrition, physical activity and ovarian cancer	103	2018	Ovarian cancer	dairy, milk	ovarian cancer incidence						milk and dairy products	
Diet, nutrition, physical activity and prostate cancer	104	2018	Prostate cancer	dairy	prostate cancer					dairy - increased risk		
Diet, nutrition, physical activity and bladder cancer	105	2018	Bladder cancer	milk, yogurt, cheese	bladder cancer incidence						for milk, yoghurt, cheese	
Diet, nutrition, physical activity and kidney cancer	107	2018	Kidney cancer	milk and dairy	kidney cancer incidence						milk and dairy products	
Diet, nutrition, physical activity and oesophageal cancer	109	2018	Oesophageal cancer	milk and dairy products	Oesophageal cancer incidence						milk and dairy products	
Diet, nutrition, physical activity and breast cancer	110	2018	Breast cancer	dairy	breast cancer incidence					dairy products for premenopausal cancer decreases risk	dairy products for post-menopausal cancer	
Diet, nutrition, physical activity and colorectal cancer	111	2018	Colorectal cancer	dairy	colorectal cancer			dairy products - decreased risk				
Diet, nutrition, physical activity and lung cancer	112	2018	Lung cancer	milk and dairy products	lung cancer						milk and dairy products	
Diet, nutrition, physical activity and cancers of the mouth, pharynx and larynx	113	2018	Cancers of the mouth, pharynx, and larynx	dairy	mouth, pharynx and larynx cancer						dairy products	

Supplemental Table C: Excluded papers

Title, First Author, Year	Reason for Exclusion
A Meta-Analysis of Randomized Clinical Trials on Calcium Intake for Obesity Indices (Hong, 2021)	Other
A Prospective Diet-Wide Association Study for Risk of Colorectal Cancer in EPIC (Papadimitriou, 2022)	Other
A systematic review of the effect of yogurt consumption on chronic diseases risk markers in adults (Dumas, 2017)	Concept: enriched/supplemented dairy products
A systematic review on the effectiveness of diet and exercise in the management of obesity (Olateju, 2023)	Other
Acne and Nutrition: A Systematic Review (Fiedler, 2017)	wrong health outcome
Association between animal protein sources and risk of neurodegenerative diseases: a systematic review and dose-response meta-analysis (Talebi, 2023)	wrong health outcome
Association between dietary pattern and risk of cardiovascular disease among adults in the Middle East and North Africa region: a systematic review (Aljefree, 2015)	Other
Association of dairy products consumption with risk of obesity in children and adults: a meta-analysis of mainly cross-sectional studies (Wang, 2016)	Population: infants, children, or adolescents
Associations between food and beverage groups and major diet-related chronic diseases: an exhaustive review of pooled/ meta-analyses and systematic reviews (Fardet, 2013)	Type of evidence: umbrella review
Associations between potential causal factors and colorectal cancer risk: A systematic review and meta-analysis of Mendelian randomization studies (Deng, 2021)	Type of evidence: mendellian randomization
Associations between Yogurt Consumption and Weight Gain and Risk of Obesity and Metabolic Syndrome: A Systematic Review (Sayon-Orea, 2016)	wrong health outcome
Associations of Dietary Intake with Cardiovascular Disease, Blood Pressure, and Lipid Profile in the Korean Population: a Systematic Review and Meta-Analysis (Kim, 2020)	Other
Benefits of Probiotic Yogurt Consumption on Maternal Health and Pregnancy Outcomes: A Systematic Review (He, 2020)	Concept: enriched/supplemented dairy products
Calcium and dairy products in the chemoprevention of colorectal adenomas: a systematic review and meta-analysis (Emami, 2021)	Concept: nutritional supplements as exposure

Calcium intake and risk of fracture: systematic review (Bolland, 2014)	Concept: nutritional supplements as exposure
Consumption of Dairy Foods and Cardiovascular Disease: A Systematic Review (Giosuè, 2022)	Type of evidence: umbrella review
Consumption of Dairy Products in Relation to Changes in Anthropometric Variables in Adult Populations: A Systematic Review and Meta-Analysis of Cohort Studies (Schwingshackl, 2016)	wrong health outcome
Dairy Consumption and Cardiometabolic Diseases: Systematic Review and Updated Meta-Analyses of Prospective Cohort Studies (Soedamah-Muthu, 2018)	Other
Dairy consumption and insulin sensitivity: A systematic review of short-and long-term intervention studies (Turner, 2015)	wrong health outcome
Dairy Consumption and Risk of Conventional and Serrated Precursors of Colorectal Cancer: A Systematic Review and Meta-Analysis of Observational Studies (Guo, 2021)	wrong health outcome
Dairy consumption and risk of metabolic syndrome: a meta-analysis (Kim, 2016)	wrong health outcome
Dairy Consumption and Risk of Metabolic Syndrome: Results from Korean Population and Meta-Analysis (Jin, 2021)	wrong health outcome
Dairy consumption and risk of testicular cancer: a systematic review (Signal, 2018)	wrong health outcome
Dairy Consumption and Total Cancer and Cancer-Specific Mortality: A Meta-Analysis of Prospective Cohort Studies (Shaoyue, 2022)	wrong health outcome
Dairy food consumption is associated with a lower risk of the metabolic syndrome and its components: a systematic review and meta-analysis (Lee, 2018)	wrong health outcome
Dairy Foods and Dairy Proteins in the Management of Type 2 Diabetes: A Systematic Review of the Clinical Evidence 1,2 (Pasin, 2015)	Concept: nutritional supplements as exposure
Dairy foods, calcium, and risk of breast cancer overall and for subtypes defined by estrogen receptor status: a pooled analysis of 21 cohort studies (Wu, 2021)	Concept: nutritional supplements as exposure
Dairy intake and acne development: A meta-analysis of observational studies (Aghasi, 2019)	Population: infants, children, or adolescents
Dairy intake and bone health across the lifespan: a systematic review and expert narrative (Wallace, 2021)	Population: infants, children, or adolescents
Dairy product consumption and development of cancer: an overview of reviews (Jeyaraman, 2019)	Type of evidence: umbrella review
Dairy product consumption and risk of non-alcoholic fatty liver disease: A systematic review and meta-analysis of observational studies (Yuzbashian, 2021)	wrong health outcome

Dairy Product Consumption in the Prevention of Metabolic Syndrome: A Systematic Review and Meta-Analysis of Prospective Cohort Studies (Mena-Sánchez, 2019)	wrong health outcome
Dairy Product, Calcium Intake and Lung Cancer Risk: A Systematic Review with Meta-Analysis (Yang, 2016)	Concept: nutritional supplements as exposure
Dairy products and chronic kidney disease: protective or harmful? a systematic review of prospective cohort studies (Eslami, 2018)	wrong health outcome
Dairy products and colorectal cancer in middle eastern and north African countries: a systematic review (El Kinany, 2018)	Concept: nutritional supplements as exposure
Dairy products and inflammation: A review of the clinical evidence (Bordoni, 2017)	Population: infants, children, or adolescents
Dairy products and inflammation: A review of theclinical evidence (Bordoni, 2014)	wrong health outcome
Dairy products and pancreatic cancer risk: a pooled analysis of 14 cohort studies (Genkinger, 2014)	Population: infants, children, or adolescents
Dairy products consumption and metabolic syndrome in adults: systematic review and metaanalysis of observational studies (Chen, 2015)	wrong health outcome
Dairy products intake and cancer mortality risk: a meta-analysis of 11 population-based cohort studies (Lu, 2016)	wrong health outcome
Dairy products, calcium, and prostate cancer risk: a systematic review and meta-analysis of cohort studies (Aune, 2015)	Concept: nutritional supplements as exposure
Dairy Products: Is There an Impact on Promotion of Prostate Cancer? A Review of the Literature (Vasconelos, 2019)	Type of evidence: umbrella review
Diet and acne: A systematic review (Meixiong, 2022)	Population: infants, children, or adolescents
Diet and acne: review of the evidence from 2009 to 2020 (Dall'Oglio, 2021)	Population: infants, children, or adolescents
Diet and risk of diabetic retinopathy: a systematic review (Dow, 2018)	Concept: Dairy not from cows, formula products, or non-dairy
Dietary and metabolic factors in the pathogenesis of hidradenitis suppurativa: a systematic review (Choi, 2020)	Concept: Dairy not from cows, formula products, or non-dairy
Dietary Consumption on Glycemic Control Among Prediabetes: A Review of the Literature (Thipsawat, 2023)	Concept: Dairy not from cows, formula products, or non-dairy
Dietary factors and onset of natural menopause: A systematic review and meta-analysis (Grisotto, 2021)	Other

Dietary factors and risk of gout and hyperuricemia: a meta-analysis and systematic review (Li, 2018)	wrong health outcome
Dietary Patterns in Relation to Low Bone Mineral Density and Fracture Risk: A Systematic Review and Meta-Analysis (Fabiani, 2019)	Population: infants, children, or adolescents
Dietary Protein Consumption and the Risk of Type 2 Diabetes: ADose-Response Meta-Analysis of Prospective Studies (Fan, 2019)	Other
Dietary protein intake and prostate cancer risk in adults: A systematic review and dose-response meta-analysis of prospective cohort studies (Alzahrani, 2022)	Other
Do alcoholic beverages, obesity and other nutritional factors modify the risk<br>of familial colorectal cancer? A systematic review (Fardet, 2017)	Concept: wrong exposure
Effect of cheese consumption on blood lipids: a systematic review and meta-analysis of randomized controlled trials (De Goede, 2015)	wrong health outcome
Effect of daily probiotic yogurt consumption on inflammation: A systematic review and meta-analysis of randomized Controlled Clinical trials (Mousavi, 2020)	Concept: enriched/supplemented dairy products
Effect of dairy intake with or without energy restriction on body composition of adults: overview of systematic reviews and meta-analyses of randomized controlled trials (Lopez-Sobaler, 2020)	wrong health outcome
Effect of increasing dietary calcium through supplements and dairy food on body weight and body composition: a meta-analysis of randomised controlled trials (Booth, 2015)	Concept: enriched/supplemented dairy products
Effect of kefir beverage consumption on glycemic control: A systematic review and meta-analysis of randomized controlled clinical trials (Salari, 2021)	wrong health outcome
Effect of Milk and Other Dairy Products on the Risk of Frailty, Sarcopenia, and Cognitive Performance Decline in the Elderly: A Systematic Review (Cuesta-Triana, 2019)	wrong health outcome
Effect of probiotic foods and supplements on blood pressure: a systematic review of meta-analyses studies of controlled trials (Ejtahed, 2020)	Population: infants, children, or adolescents
Effect of probiotic yogurt on gestational diabetes mellitus: A systematic review and meta-analysis (Tabatabaeizadeh, 2023)	Concept: enriched/supplemented dairy products
Effects of dairy products consumption on inflammatory biomarkers among adults: A systematic review and meta-analysis of randomized controlled trials (Moosavian, 2020)	wrong health outcome
Effects of dairy products on bone mineral density in healthy postmenopausal women: a systematic review and meta-analysis of randomized controlled trials (Shi, 2020)	Concept: Dairy not from cows, formula products, or non-dairy

Effects of fermented dairy products on inflammatory biomarkers: A meta-analysis (Zhang, 2023)	wrong health outcome
Effects of fermented dairy products on inflammatory biomarkers: A meta-analysis (Zhang, 2023)	wrong health outcome
Effects of Kefir Consumption on Cardiometabolic Risk Factors: A Systematic Review and Meta-analysis of Randomized Controlled Trials (Yahyapoor, 2023)	Other
Effects of Milk and Dairy Product Consumption on Type 2 Diabetes: Overview of Systematic Reviews and Meta-Analyses (Alvarez-Bueno, 2019)	Type of evidence: umbrella review
Effects of probiotics consumption on lowering lipids and CVD risk factors: A systematic review and meta-analysis of randomized controlled trials (Sun, 2015)	Concept: nutritional supplements as exposure
Effects of probiotics fermented milk products on obesity measure among adults: A systematic review and meta-analysis of clinical trials (Mohammadi, 2021)	Concept: enriched/supplemented dairy products
Efficacy of Probiotics in Patients of Cardiovascular Disease Risk: A Systematic Review and Meta-Analysis Author (Dixon, 2020)	Concept: nutritional supplements as exposure
Environmental etiology of gastric cancer in Iran: a systematic review focusing on drinking water, soil, food, radiation, and geographical conditions (Ghaffari, 2019)	Concept: Dairy not from cows, formula products, or non-dairy
Epidemiologic Study of Gastric Cancer in Iran: A Systematic Review (Farmanfarma, 2020)	Concept: Dairy not from cows, formula products, or non-dairy
Estimation and Prediction of Avoidable Health Care Costs of Cardiovascular Diseases and Type 2 Diabetes Through Adequate Dairy Food Consumption: A Systematic Review and Micro Simulation Modeling Study (Javanbakht, 2018)	Population: infants, children, or adolescents
Etiologic effects and optimal intakes of foods and nutrients for risk of cardiovascular diseases and diabetes: Systematic reviews and meta-analyses from the Nutrition and Chronic Diseases Expert Group (NutriCoDE) (Micha, 2017)	Type of evidence: umbrella review
Evidence Update on the Relationship between Diet and the Most Common Cancers from the European Prospective Investigation into Cancer and Nutrition (EPIC) Study: A Systematic Review (Ubago-Guisado, 2021)	Other
Fermented Dairy Products, Probiotic Supplementation, and Cardiometabolic Diseases: A Systematic Review and Meta-analysis (Companys, 2020)	Concept: enriched/supplemented dairy products
Fermented Food and Non-Communicable Chronic Diseases: A Review (Gille, 2018)	Type of evidence: umbrella review

Food groups and intermediate disease markers: a systematic review and network meta-analysis of randomized trials (Schwingshackl, 2018)	wrong health outcome
Foods and beverages and colorectal cancer risk: a systematic review and meta-analysis of cohort studies, an update of the evidence of the WCRF-AICR Continuous Update Project (Vieira, 2017)	Other
Functional foods modulating inflammation and metabolism in chronic diseases: a systematic review (Luvián-Morales,2022)	Concept: enriched/supplemented dairy products
Gender-specific risk factors for gout: a systematic review of cohort studies (Evans, 2019)	wrong health outcome
Health-Promoting Components in Fermented Foods: An Up-to-Date Systematic Review (Melini, 2019)	Concept: wrong exposure
Increased Dairy Product Intake Modifies Plasma Glucose Concentrations and Glycated Hemoglobin: A Systematic Review and Meta-Analysis of Randomized Controlled Trials (O'Connor, 2019)	Population: infants, children, or adolescents
Influence of food or food groups intake on the occurrence and / or protection of different types of cancer: systematic review (Zaragoza Martí, 2019)	Other
Is dairy consumption associated with depressive symptoms or disorders in adults? A systematic review of observational studies (Hockey, 2020)	wrong health outcome
Is milk and dairy intake a preventive factor for elderly cognition (dementia and Alzheimer's)? A quality review of cohort surveys (Bermejo-Pareja, 2021)	Type of evidence: narrative review
Is replacing red meat with other protein sources associated with lower risks of coronary heart disease and all-cause mortality? A meta-analysis of prospective studies (Hidayat, 2022)	Other
Japanese-Style Diet and Cardiovascular Disease Mortality: A Systematic Review and Meta-Analysis of Prospective Cohort Studies (Shirota, 2022)	Other
Maternal consumption of dairy products and gestational weight gain, gestational diabetes mellitus, and preeclampsia: A systematic review (Huang, 2023)	wrong health outcome
Milk and Dairy Product Consumption and Cardiovascular Diseases: An Overview of Systematic Reviews and Meta-Analyses (Fontecha, 2019)	Type of evidence: umbrella review
Milk and Dairy Product Consumption and Inflammatory Biomarkers: An Updated Systematic Review of Randomized Clinical Trials (Ulven, 2019)	wrong health outcome

Milk and Dairy Product Consumption and Prostate Cancer Risk and Mortality: An Overview of Systematic Reviews and Meta-analyses (López-Plaza, 2019)	Type of evidence: umbrella review
Milk and Dairy Product Consumption and Risk of Mortality: An Overview of Systematic Reviews and Meta-Analyses (Cavero-Redondo, 2019)	Type of evidence: umbrella review
Milk Consumption and Prostate Cancer: A Systematic Review (Sargsyan, 2021)	Other
Myoprotective Whole Foods, Muscle Health and Sarcopenia: A Systematic Review of Observational and Intervention Studies in Older Adults (Granic, 2020)	wrong health outcome
No adverse effects of dairy products on lipid profile: A systematic review and meta-analysis of randomized controlled clinical trials (Derakhshandeh-Rishehri 2021)	wrong health outcome
Poor dietary intake of nutrients and food groups are associated with increased risk of periodontal disease among community dwelling older adults: a systematic literature review (O'Connor, 2020)	wrong health outcome
Potential of favorable effects of probiotics fermented milk supplementation on blood pressure: a systematic review and meta-analysis (Ghavami, 2020)	Concept: enriched/supplemented dairy products
Prostate cancer and the influence of dietary factors and supplements: a systematic review (Mandair, 2014)	Other
Risk factors for the development of lung cancer among never smokers: A systematic review (Zhang, 2022)	Other
Stroke and food groups: an overview of systematic reviews and meta-analyses (Deng, 2018)	Type of evidence: umbrella review
Systematic Review of the Association between Dairy Product Consumption and Risk of Cardiovascular-Related Clinical Outcomes (Drouin-Chartier, 2016)	Type of evidence: umbrella review
The Association between Dairy Products Consumption and Arterial Stiffness: A Meta-Analysis (Álvarez-Bueno, 2018)	wrong health outcome
The association between dietary patterns and risk of miscarriage: a systematic review and meta-analysis (Chung, 2023)	wrong health outcome
The association between milk consumption and the metabolic syndrome: a cross-sectional study of the residents of Suzhou, China and a meta-analysis (Hidayat, 2020)	wrong health outcome
The Association between Total Protein, Animal Protein, and Animal Protein Sources with Risk of Inflammatory Bowel Diseases: A Systematic Review and Meta-Analysis of Cohort Studies (Talebi, 2023)	wrong health outcome

The Effect of Dairy Products Subgroups Consumption on the Risk of Diabetes: A Systematic Review and Meta-Analysis (Khoramda, 2017)	Other
The effect of milk consumption on acne: a meta-analysis of observational studies (Dai, 2018)	Population: infants, children, or adolescents
The effect of probiotic fermented milk products on blood lipid concentrations: A systematic review and meta-analysis of randomized controlled trials (Ziaei, 2021)	wrong health outcome
The Effects of Dairy Product and Dairy Protein Intake on Inflammation: A Systematic Review of the Literature (Nieman, 2021)	wrong health outcome
The effects of kefir consumption on human health: a systematic review of randomized controlled trials (Kairey, 2023)	Population: infants, children, or adolescents
The Effects of Probiotics on Cholesterol Levels in Patients With Metabolic Syndrome: A Systematic Review (Momin, 2023)	Concept: nutritional supplements as exposure
The Impact of Prebiotic, Probiotic, and Synbiotic Supplements and Yogurt Consumption on the Risk of Colorectal Neoplasia among Adults: A Systematic Review (Kim, 2022)	Concept: nutritional supplements as exposure
The impact of probiotic yogurt consumption on lipid profiles in subjects with mild to moderate hypercholesterolemia: A systematic review and meta-analysis of randomized controlled trials (Pourrajab, 2020)	Concept: enriched/supplemented dairy products
The National Osteoporosis Foundation's position statement on peak bone mass development and lifestyle factors: a systematic review and implementation recommendations (Weaver, 2016)	Population: infants, children, or adolescents
The role of calcium and vitamin D dietary intake on risk of colorectal cancer: systematic review and meta-analysis of case-control studies (Lopez-Caleya, 2022)	Concept: wrong exposure
The role of nutrition on Parkinson's disease: a systematic review (Bianchi, 2023)	wrong health outcome
The Role of Probiotic Lactic Acid Bacteria and Bifidobacteria in the Prevention and Treatment of Inflammatory Bowel Disease and Other Related Diseases: A Systematic Review of Randomized Human Clinical Trials (Saez-Lara, 2015)	Concept: nutritional supplements as exposure
Total Dairy, Cheese and Milk Intake and Arterial Stiffness: A Systematic Review and Meta-analysis of Cross-Sectional Studies (Diez-Fernández, 2019)	Population: infants, children, or adolescents
Yogurt, cultured fermented milk, and health: a systematic review (Savaiano, 2021)	Population: infants, children, or adolescents

Supplemental Table D: Papers charted by health outcome

Health outcome	number of papers	Researcher-assigned Paper IDs												
**Cardiovascular outcomes**														
CVD	**8**	67	6	47	12	10	3	16	86					
CHD	**11**	11	12	58	67	79	6	16	24	19	51	86		
Stroke	**12**	19	58	11	12	21	79	24	6	16	3	51	86	
Hypertension/HBP	**5**	8	28	24	52	35								
**Cancers**														
Bladder	**6**	44	85	59	68	38	105							
Breast	**8**	60	70	83	31	82	88	110	72					
Colorectal	**10**	4	92	76	46	38	40	56	50	72	111			
Corpus uteri	**2**	30	102											
Esophagus	**3**	18	109	38										
Kidney	**1**	107												
Leukemia	**1**	65												
Liver	**3**	14	80	43										
Lung	**2**	15	112											
Non-Hodgkin lymphoma	**2**	27	55											
Oral	**3**	57	69	113										
Ovarian	**3**	73	36	103										
Pancreatic	**1**	23												
Prostate	**3**	84	104	72										
Stomach/gastric	**5**	2	13	25	74	89								
Cancer (any)	**1**	38												
**Body Composition**														
Overweight	**2**	8	53											
Obesity	**2**	8	53											
Weight gain	**4**	61	87	53	41									
**Mortality**														
All-cause mortality	**9**	11	29	42	71	5	90	91	49	94				
CV mortality	**6**	67	71	90	91	63	94							
**Others**														
Type 2 Diabetes Mellitus	**8**	7	8	34	20	54	33	3	99					
Cognitive outcomes	**3**	78	66	22										
Bone health	**4**	37	9	26	48									
Joint health	**2**	77	1											

Supplemental Table E: Papers charted by exposure

	Milk Paper ID*	Cheese Paper ID	Yogurt Paper ID	All Dairy Paper ID	All fermented dairy paper ID
**Total # of papers:**	**52**	**42**	**40**	**66**	**13**
	2	2	2	1	4
	4	4	4	2	8
	7	5	6	3	19
	8	6	7	4	28
	9	8	10	7	33
	11	12	12	8	34
	12	14	14	9	38
	13	16	18	12	40
	14	18	19	13	42
	18	19	23	14	47
	19	20	24	15	67
	21	21	26	16	68
	22	22	27	18	88
	23	23	28	19	
	24	24	31	20	
	25	26	33	21	
	26	27	34	22	
	27	28	37	24	
	28	30	38	25	
	30	34	43	26	
	37	36	46	28	
	42	37	47	30	
	43	38	48	31	
	44	40	56	34	
	57	42	57	35	
	58	43	58	36	
	59	46	59	37	
	60	47	60	43	
	66	48	61	44	
	67	57	67	49	
	68	58	70	50	
	69	59	73	51	
	70	60	76	52	
	71	67	80	53	
	72	68	83	54	
	73	76	84	55	
	76	79	90	58	

	78	80	91	63	
	79	83	99	65	
	80	84	105	67	
	83	92		68	
	84	105		69	
	85			74	
	92			76	
	99			77	
	102			78	
	103			80	
	105			82	
	109			83	
	107			84	
	112			86	
	94			87	
				88	
				89	
				92	
				99	
				102	
				103	
				104	
				109	
				107	
				110	
				111	
				112	
				113	
				94	

*All paper-IDs are researcher-assigned. See Included Papers extraction sheet for more information

Supplemental Table F: Charting of author-assigned paper-IDs in Table 1

Health outcome	All Dairy				Milk				Cheese				Yogurt				All fermented dairy			
	↓	ø	↑	↔	↓	ø	↑	↔	↓	ø	↑	↔	↓	ø	↑	↔	↓	ø	↑	↔
**Cardiovascular outcomes**																				
**CVD***	3,16,86	12,67				67			6,47,67	12			47	10,12,67			47,67			
**CHD***	24	12,16,19,51,58,67,86				11,12,58,67,79			6,12,16, 58	67,79		24		12,58,67		24		67		
**Stroke**	3,12,16,21,24,51,86	19			19,58	11,12,21,58,79		24	6,12,16,19,21, 79	58		24		19,58		24	19			
**Hypertension/ HBP***	8,28,52,24	35			8,28			24		8,28		24		8,28		24	28	8		
**Cancers**																				
**Bladder**	44	68			44,85	59,105		68		59,68,105			59,38	105			68,38			
**Breast**	82,88,83	31,110				60,70,83	60		60	83			31,83	60,70			88			
**Colorectal**	4,50,76,92,111				4,92	76			4,46,38	40,76,92			4,56,38	46		76	38	4,40		
**Corpus uteri**		30,102				30,102				30										
**Esophagus**		18,109				18,109				18			18				38			
**Kidney**		107				107														
**Leukemia**		65																		
**Liver**		14,43	80			14,80	43			14,43		80	14,43	80						
**Lung**		15,112				112														
**Non-Hodgkin lymphoma**			55				27			27				27						
**Oral**	69	113			57,69				57				57							
**Ovarian**		103	36			73,103			36					73						

**Pancreatic**						23				23				23						
**Prostate**		104	84			72	84				84			84						
**Stomach/gastric**		2,25	74	13		2,13		25		2										
**All**																	38			
**Body composition**																				
**Overweight**	8	53			8									8						
**Obesity**	8	53			8									8						
**Weight gain**	87	53											61							
**Mortality**																				
**All-cause mortality**		49, 67, 94				11,42, 67, 94		71		5,42, 67			90,91	67			67		42	
**CV* mortality**		63		94		94		71					90, 91							
**Others**																				
**Type 2 Diabetes Mellitus**	3,7,8, 34,54, 99	20			99	7,8,34				8,34	20		7,8,33, 34,99				33,34			
**Joint health**	77	1																		
**Bone health**				9,26, 37		37		9,26	37			26,48	37,48			26				
**Cognitive health**		78		22		78		66,22				22								

*CVD, cardiovascular disease; CHD, coronary heart disease; HBP, high blood pressure; CV, cardiovascular.

Supplementary File G: Ovid MEDLINE Search Strategy run on February 19, 2024

1	exp Dairy Products/
2	(dairy or milk or butter$ or ghee or cheese$ or cream$ or yogurt$ or yoghurt$ or kefir$ or buttermilk or koumiss).ti,kf. or (dairy or milk or butter$ or ghee or cheese$ or cream$ or yogurt$ or yoghurt$ or kefir$ or buttermilk or koumiss).ab. /freq=2
3	1 or 2
4	Bone Density/
5	Alzheimer Disease/
6	Dementia/
7	exp Inflammation/
8	exp Multiple Sclerosis/
9	exp Acne Vulgaris/
10	exp diabetes mellitus, type 2/
11	Prediabetic State/
12	exp insulin resistance/
13	hyperglycemia/
14	dyslipidemias/
15	hyperlipidemias/
16	hypercholesterolemia/
17	exp hypertriglyceridemia/
18	mortality/
19	exp neoplasms/
20	metabolic syndrome/
21	exp hypertension/
22	cardiovascular diseases/
23	heart diseases/
24	vascular diseases/
25	exp Stroke/
26	exp Arteriosclerosis/
27	overweight/
28	obesity/
29	obesity, abdominal/
30	obesity, morbid/
31	body weight changes/
32	weight gain/
33	weight loss/
34	or/5-33
35	(bone density or bone densities or bone mineral content or bone mineral contents).ti,ab,kf.
36	(bone adj3 (frailty or fragility)).ti,ab,kf.
37	alzheimer*.ti,ab,kf.
38	(dementia or dementias).ti,ab,kf.
39	((inflamm$ or anti-inflamm$ or metabolic$ or cardiometabolic or cardiovascular) adj5 (syndrome$ or disorder$ or outcome$ or biomarker$)).ti,ab,kf.
40	multiple sclerosis.ti,ab,kf.
41	acne.ti,ab,kf.
42	hypertens$.ti,ab,kf.
43	((high$ or increas$ or elevat$ or low$) adj5 blood pressure).ti,ab,kf.
44	((cardiovascul$ or cardiac$ or heart or coronary or myocard$ or pericard$ or vascular or artery or arteries or arterial or vessel or vessels) adj3 (disease$ or disorder$)).ti,ab,kf.

45	Arteriosclero$.ti,ab,kf.
46	(adipos$ or obese$ or obesit$ or overweight).ti,ab,kf.
47	((body mass index or bmi or fat mass or (body adj2 fat) or body composition$ or anthropometr$) adj5 (change$ or differ$ or reduc$ or low$ or increas$ or gain$ or elevat$)).ti,ab,kf.
48	(stroke or isch?em$ or cerebrovasc$ or apoplexy or ((brain$ or cerebral or lacunar) adj2 infarct$)).ti,ab,kf.
49	(prediabet$ or pre-diabet$).ti,ab,kf.
50	insulin resistan$.ti,ab,kf.
51	(dm2 or t2d or dm type 2 or type 2 diabet* or dm type II or type two diabet* or type II diabet* or dm type II).ti,ab,kf.
52	hyperglycemi$.ti,ab,kf.
53	HbA1c.ti,ab,kf.
54	(dyslipid?emia$ or Hyperlip?emia$ or Hyperlipid?emia$ or Lipide?mia$ or Lipe?mia$).ti,ab,kf.
55	(Hypercholesterolem$ or Hypercholester?emia$ or ((high or increas$ or elevat$ or low$) adj5 cholesterol$)).ti,ab,kf.
56	hypertriglycerid?emia$.ti,ab,kf.
57	mortality.ti,ab,kf.
58	(cancer$ or carcinoma$ or adenocarcin$ or tumor$ or tumour$ or neoplasm$ or neoplastic or neoplasia or malignan$ or metastases or metastasis or metastatic or carcinoid$ or oncol$).ti,ab,kf.
59	or/35-58
60	34 or 59
61	3 and 60
62	(Adolescent/ or Child/ or Infant/ or adolescen*.ti,ab,kf. or child*.ti,ab,kf. or schoolchild*.ti,ab,kf. or infant*.ti,ab,kf. or girl*.ti,ab,kf. or boy*.ti,ab,kf. or teen.ti,ab,kf. or teens.ti,ab,kf. or teenager*.ti,ab,kf. or youth*.ti,ab,kf. or pediatr*.ti,ab,kf. or paediatr*.ti,ab,kf. or puber*.ti,ab,kf.) not (Adult/ or adult*.ti,ab,kf. or man.ti,ab,kf. or men.ti,ab,kf. or woman.ti,ab,kf. or women.ti,ab,kf.)
63	61 not 62
64	exp animals/ not humans.sh.
65	63 not 64
66	(((systematic* and review?) or Systematic overview* or ((Cochrane or systemic or scoping or mapping or Umbrella) adj review*) or ((Cochrane or systemic or scoping or mapping or Umbrella) adj literature review*) or "review of reviews" or "overview of reviews" or meta-review or (integrat* adj (review or overview)) or meta-synthes?s or metasynthes?s or "quantitative review" or "quantitative synthesis" or "research synthesis" or meta-ethnography or "Systematic literature search" or "Systematic literature research" or meta-analys?s or metaanalys?s or "meta-analytic review" or "meta-analytical review").ti,kf,bt. or meta-analysis.pt. or Network Meta-Analysis/ or ((search* or medline or pubmed or embase or Cochrane or scopus or "web of science" or "sources of information" or "data sources" or "following databases") and ("study selection" or "selection criteria" or "eligibility criteria" or "inclusion criteria" or "exclusion criteria")).tw. or "systematic review".pt.) not ((letter or editorial or comment or "case reports" or "historical article").pt. or report.ti. or protocol.ti. or protocols.ti. or withdrawn.ti. or "retraction of publication".pt. or exp "retraction of publication as topic"/ or "retracted publication".pt. or reply.ti. or "published erratum".pt.)
67	65 and 66
68	limit 67 to yr="2014 -Current"

Supplementary File H: Ovid Embase Search Strategy, 2014 to present, run on February 19, 2024

1	exp Dairy Product/
2	(dairy or milk or butter* or ghee or cheese* or cream* or yog?urt* or kefir* or buttermilk or koumiss).ti,kf. or (dairy or milk or butter* or ghee or cheese* or cream* or yog?urt* or kefir* or buttermilk or koumiss).ab. /freq=2
3	1 or 2
4	Bone Density/
5	Alzheimer Disease/
6	Dementia/
7	exp Inflammation/
8	exp Multiple Sclerosis/
9	exp Acne Vulgaris/
10	exp non insulin dependent diabetes mellitus/
11	Impaired glucose tolerance/
12	exp insulin resistance/
13	hyperglycemia/
14	dyslipidemia/
15	hyperlipidemia/
16	hypercholesterolemia/
17	exp hypertriglyceridemia/
18	mortality/
19	exp neoplasm/
20	metabolic syndrome x/
21	exp hypertension/
22	cardiovascular disease/
23	heart disease/
24	vascular disease/
25	exp cerebrovascular accident/
26	exp Arteriosclerosis/
27	overweight/
28	obesity/
29	Abdominal obesity/
30	Morbid obesity/
31	body weight change/
32	Body weight gain/
33	Body weight loss/
34	or/5-33
35	(bone density or bone densities or bone mineral content or bone mineral contents).ti,ab,kf.
36	(bone adj3 (frailty or fragility)).ti,ab,kf.
37	alzheimer*.ti,ab,kf.
38	(dementia or dementias).ti,ab,kf.
39	((inflamm* or anti-inflamm* or metabolic* or cardiometabolic or cardiovascular) adj5 (syndrome* or disorder* or outcome* or biomarker*)).ti,ab,kf.
40	multiple sclerosis.ti,ab,kf.
41	acne.ti,ab,kf.
42	hypertens*.ti,ab,kf.
43	((high* or increas* or elevat* or low*) adj5 blood pressure).ti,ab,kf.
44	((cardiovascul* or cardiac* or heart or coronary or myocard* or pericard* or vascular or artery or arteries or arterial or vessel or vessels) adj3 (disease* or disorder*)).ti,ab,kf.

45	Arteriosclero*.ti,ab,kf.
46	(adipos* or obese* or obesit* or overweight).ti,ab,kf.
47	((body mass index or bmi or fat mass or (body adj2 fat) or body composition* or anthropometr*) adj5 (change* or differ* or reduc* or low* or increas* or gain* or elevat*)).ti,ab,kf.
48	(stroke or isch?em* or cerebrovasc* or apoplexy or ((brain* or cerebral or lacunar) adj2 infarct*)).ti,ab,kf.
49	(prediabet* or pre-diabet*).ti,ab,kf.
50	insulin resistan*.ti,ab,kf.
51	(dm2 or t2d or dm type 2 or type 2 diabet* or dm type II or type two diabet* or type II diabet* or dm type II).ti,ab,kf.
52	Hyperglyc?emi*.ti,ab,kf.
53	HbA1c.ti,ab,kf.
54	(dyslipid?emia* or Hyperlip?emia* or Hyperlipid?emia* or Lipide?mia* or Lipe?mia*).ti,ab,kf.
55	(Hypercholesterolem* or Hypercholester?emia* or ((high or increas* or elevat* or low*) adj5 cholesterol*)).ti,ab,kf.
56	hypertriglycerid?emia*.ti,ab,kf.
57	mortality.ti,ab,kf.
58	(cancer* or carcinoma* or adenocarcin* or tumor* or tumour* or neoplasm* or neoplastic or neoplasia or malignan* or metastases or metastasis or metastatic or carcinoid* or oncol*).ti,ab,kf.
59	or/35-58
60	34 or 59
61	3 and 60
62	(Adolescent/ or Child/ or Infant/ or adolescen*.ti,ab,kf. or child*.ti,ab,kf. or schoolchild*.ti,ab,kf. or infant*.ti,ab,kf. or girl*.ti,ab,kf. or boy*.ti,ab,kf. or teen.ti,ab,kf. or teens.ti,ab,kf. or teenager*.ti,ab,kf. or youth*.ti,ab,kf. or pediatr*.ti,ab,kf. or paediatr*.ti,ab,kf. or puber*.ti,ab,kf.) not (Adult/ or adult*.ti,ab,kf. or man.ti,ab,kf. or men.ti,ab,kf. or woman.ti,ab,kf. or women.ti,ab,kf.)
63	61 not 62
64	exp animal/ not human.sh.
65	63 not 64
66	(((systematic* and review?) or Systematic overview* or ((Cochrane or systemic or scoping or mapping or Umbrella) adj review*) or ((Cochrane or systemic or scoping or mapping or Umbrella) adj literature review*) or "review of reviews" or "overview of reviews" or meta-review or (integrat* adj (review or overview)) or meta-synthes?s or metasynthes?s or "quantitative review" or "quantitative synthesis" or "research synthesis" or meta-ethnography or "Systematic literature search" or "Systematic literature research" or meta-analys?s or metaanalys?s or "meta-analytic review" or "meta-analytical review").ti,kf,bt. or meta-analysis.pt. or Network Meta-Analysis/ or ((search* or medline or pubmed or embase or Cochrane or scopus or "web of science" or "sources of information" or "data sources" or "following databases") and ("study selection" or "selection criteria" or "eligibility criteria" or "inclusion criteria" or "exclusion criteria")).tw. or "systematic review".pt.) not ((letter or editorial or comment or "case reports" or "historical article").pt. or report.ti. or protocol.ti. or protocols.ti. or withdrawn.ti. or "retraction of publication".pt. or exp "retraction of publication as topic"/ or "retracted publication".pt. or reply.ti. or "published erratum".pt.)
67	65 and 66
68	limit 67 to yr="2014 -Current"

Supplementary File I: Web of Science Search Strategy, Searched on February 19, 2024

1	ALL="Dairy Products"
2	TS=(dairy or milk or butter* or ghee or cheese* or cream* or yog$urt* or kefir* or buttermilk or koumiss)
3	#1 or #2
4	TS="Bone Density"
5	TS="Alzheimer* Disease"
6	TS=Dementia
7	TS=Inflammation
8	TS="Multiple Sclerosis"
9	TS="Acne Vulgaris"
10	TS="type 2 diabetes mellitus"
11	TS="Prediabetic State"
12	TS="insulin resistance"
13	TS=hyperglyc$emia
14	TS=dyslipidemias
15	TS=hyperlipidemias
16	TS=hypercholesterolemia
17	TS=hypertriglyceridemia
18	TS=mortality
19	TS=neoplasms
20	TS="metabolic syndrome"
21	TS=hypertension
22	TS="cardiovascular disease*"
23	TS="heart diseases"
24	TS= "vascular diseases"
25	TS=Stroke
26	TS=Arteriosclerosis
27	TS=overweight
28	TS=obesity
29	TS= "abdominal obesity"
30	TS= "morbid obesity"
31	TS= "body weight changes"
32	TS= "weight gain"
33	TS= "weight loss"
34	#5 OR #6 OR #7 OR #8 OR #9 OR #10 OR #11 OR #12 OR #13 OR #14 OR #15 OR #16 OR #17 OR #18 OR #19 OR #20 OR #21 OR #22 OR #23 OR #24 OR #25 OR #26 OR #27 OR #28 OR #29 OR #30 OR #31 OR #32 OR #33
35	TS=(bone density or bone densities or bone mineral content or bone mineral contents)
36	TS=(bone NEAR/3 (frailty or fragility))
37	TS=(alzheimer*)
38	TS=(dementia*)
39	TS=((inflamm* or anti-inflamm* or metabolic* or cardiometabolic or cardiovascular) NEAR/5 (syndrome* or disorder* or outcome* or biomarker*))
40	TS=(multiple sclerosis)
41	TS=(acne)
42	TS=(hypertens*)
43	TS=((high* or increas* or elevat* or low*) NEAR/5 (blood pressure))
44	TS=((cardiovascul* or cardiac* or heart or coronary or myocard* or pericard* or vascular or artery or arteries or arterial or vessel or vessels) NEAR/3 (disease* or disorder*))
45	TS=(Arteriosclero*)
46	TS=(adipos* or obese* or obesit* or overweight)

47	TS=((“body mass index” or bmi or “fat mass” or (body NEAR/2 fat) or “body composition*” or anthropometr*) NEAR/5 (change* or differ* or reduc* or low* or increas* or gain* or elevat*))
48	TS=(stroke or isch$em* or cerebrovasc* or apoplexy or ((brain* or cerebral or lacunar) NEAR/2 infarct*))
49	TS=(prediabet* or pre-diabet*)
50	TS=(“insulin resistan*”)
51	TS=(dm2 or t2d or “dm type 2” or “type 2 diabet*” or “dm type II” or “type two diabet*” or “type II diabet*” or “dm type II”)
52	TS=(hyperglycemi*)
53	TS=(HbA1c)
54	TS=(dyslipid$emia* or Hyperlip$emia* or Hyperlipid$emia* or Lipide$mia* or Lipe$mia*)
55	TS=(Hypercholesterolem* or Hypercholester$emia* or ((high or increas* or elevat* or low*) NEAR/5 cholesterol*))
56	TS=(Hypertriglycerid$emia*)
57	TS=(mortality)
58	TS=(cancer* or carcinoma* or adenocarcin* or tumo$r* or neoplasm* or neoplastic or neoplasia or malignan* or metasta* or carcinoid* or oncol*)
59	#35 OR #36 OR #37 OR #38 OR #39 OR #40 OR #41 OR #42 OR #43 OR #44 OR #45 OR #46 OR #47 OR #48 OR #49 OR #50 OR #51 OR #52 OR #53 OR #54 OR #55 OR #56 OR #57 OR #58
60	#34 or #59
61	#3 and #60
62	TS=((Adolescent OR Child OR Infant OR adolescen* OR child* OR schoolchild* OR infant* OR girl* OR boy* OR teen OR teens OR teenager* OR youth* OR pediatr* OR paediatr* OR puber*) NOT (Adult OR adult* OR man OR men OR woman OR women))
63	#61 not #62
64	TS=(animals) NOT TS=(humans)
65	#63 not #64
66	TS=((systematic* AND review$) OR "Systematic overview*" OR ((Cochrane OR systemic OR scoping OR mapping OR Umbrella) NEAR/0 review*) OR ((Cochrane OR systemic OR scoping OR mapping OR Umbrella) NEAR/0 "literature review*") OR "review of reviews" OR "overview of reviews" OR meta-review OR (integrat* NEAR/0 (review OR overview)) OR meta-synthes$s OR metasynthes$s OR "quantitative review" OR "quantitative synthesis" OR "research synthesis" OR meta-ethnography OR "Systematic literature search" OR "Systematic literature research" OR meta-analys$s OR metaanalys$s OR "meta-analytic review" OR "meta-analytical review")
67	TI=((“meta-analysis” OR "Network Meta-Analysis" OR "sources of information" OR "data sources" OR "following databases") AND ("study selection" OR "selection criteria" OR "eligibility criteria" OR "inclusion criteria" OR "exclusion criteria") OR ("systematic review") NOT (letter OR editorial OR comment OR "case reports" OR "historical article" OR report OR protocol OR protocols OR withdrawn OR "retraction of publication" OR "retraction of publication as topic" OR "retracted publication" OR reply OR "published erratum"))
68	#65 and #66 and #67
69	Limit #68 to intended search range OR apply a limit at the beginning.
